# Identification and prioritisation of tumour antigen candidates from 79 glioblastoma transcriptomes

**DOI:** 10.1007/s00262-026-04390-3

**Published:** 2026-04-21

**Authors:** Špela Kert, Jože Pižem, Sara Petrin, Matic Bošnjak, Miha Jerala, Alenka Matjašič, Andrej Zupan

**Affiliations:** Faculty of Medicine, Institute of Pathology, University of Ljubljana, Ljubljana, Slovenia

**Keywords:** Glioblastoma, Tumor antigens, Computational immunology, HLA class I, Transcriptome sequencing

## Abstract

Glioblastoma (GBM) is an aggressive brain tumour with limited responsiveness to current immunotherapeutic approaches, partly due to its low mutational burden and intra-tumour heterogeneity. A systematic understanding of the tumour antigen landscape is therefore essential for advancing tumour immunology and supporting rational development of immunotherapeutic strategies. In this study, we performed whole-transcriptome sequencing of RNA extracted from 79 formalin-fixed paraffin-embedded (FFPE) IDH-wildtype GBM samples to systematically identify and prioritise candidate tumour antigens derived from three sources: single-nucleotide variants (SNVs), overexpressed tumour-associated antigens (TAAs), and gene fusion events. Candidate peptides were evaluated using integrated computational criteria, including transcript expression, predicted antigen processing features, peptide–HLA binding affinity and stability. Across the cohort, mutation-derived tumor-specific antigens (TSAs) were largely private to individual samples, whereas TAAs constituted a larger and more recurrent candidate pool. Despite comparable predicted binding characteristics across antigen classes, recurrence patterns differed substantially, reflecting their distinct biological origins. Fusion-derived candidates were rare and sample-specific. Predicted peptide presentation was disproportionately associated with a limited subset of HLA class I alleles. Collectively, this study provides a systematically prioritized catalogue of transcriptionally expressed GBM antigen candidates and offers a comparative evaluation of mutation-, expression-, and fusion-derived antigen sources within a unified transcriptome-based framework.

**Supplementary Information:**

The online version contains supplementary material available at 10.1007/s00262-026-04390-3.

## Introduction

Glioblastoma (GBM) is the most common and aggressive primary brain tumour in adults and remains a major clinical challenge. While imaging techniques are important for the initial diagnosis, the final diagnosis depends on histopathological analysis and molecular genetic testing of tumour tissue. Standard treatment has remained largely unchanged for decades, consisting of maximal safe surgical resection, radiotherapy and chemotherapy with temozolomide [1]. Despite this aggressive therapy, the prognosis remains poor, with a five-year survival rate of approximately 5% [2].

Immunotherapies are emerging as an important area of investigation for the development of more precise and personalised treatments in oncology, including GBM [3–5]. Approaches leveraging antigens are often divided into two broad categories [5]. The first targets tumour-associated antigens (TAAs), which are self- proteins that are overexpressed or, such as cancer testis antigens (CTAs), abnormally expressed in malignant cells [3–6]. While TAAs can be recognised by the immune system, their expression in normal tissue may limit immune specificity and remains an important consideration for therapeutic targeting [7]. The second approach focuses on neoantigens, also referred to as tumour-specific antigens (TSAs) [7]. TSAs are novel peptides that arise from tumour-specific somatic mutations, including single nucleotide variants (SNVs), insertions, deletions, and gene fusions [8–10]. These altered peptides may be presented on the surface of tumour cells by the major histocompatibility complex and can be distinguished from self by the adaptive immune system [8–10].

Therapeutic strategies that exploit TSAs include personalised peptide or mRNA vaccines, developed from a patient’s unique tumour mutational landscape, as well as adoptive T cell approaches targeting neoantigen-specific T cells [8–10]. Preclinical and early clinical studies have demonstrated that such strategies can induce robust, tumour-specific immune responses; however, their translation into effective therapies for GBM remains challenging [8–10]. Limited efficacy has been attributed to multiple factors, including highly immunosuppressive tumour microenvironment, inter-patient variability in human leukocyte antigen (HLA) genotypes, heterogeneity in antigen presentation, and the difficulty of reliably identifying and prioritising biologically relevant antigen candidates [11, 12].

In addition, GBM has several mechanisms to evade immune recognition, such as the release of immunosuppressive cytokines and the recruitment of regulatory immune cells, which together restrict effective anti-tumour immunity [13, 14]. Genetic and clonal heterogeneity further complicates immune targeting, as tumours are composed of multiple cellular subpopulations with distinct antigenic profiles [13, 14]. Consequently, immune responses directed against antigens present only in limited tumour subclones may permit the outgrowth of antigen-negative cells, contributing to immune escape [13, 14]. To address these challenges, current strategies focus on developing vaccines targeting multiple TSAs and/or TAAs, often in combination with immune checkpoint inhibitors or other immunomodulatory agents designed to boost the T cell response and reduce tumour-mediated immune resistance [10, 15].

A major challenge in identifying TSA targets in GBM is the relatively low mutational burden of this tumour compared with more immunogenic cancers such as melanoma or non-small cell lung cancer [16]. Most TSAs in GBM are due to SNVs, which usually alter only a single amino acid, whereas insertions, deletions, and gene fusion events occur less frequently but may generate structurally distinct peptides [16]. This limited abundance of mutant TSAs highlights the importance of systematically exploring additional antigen sources, including TAAs, to better define the overall tumour antigen repertoire in GBM [17].

Recent clinical studies have shown that immune responses can be elicited against multiple tumour antigens in patients with GBM, underscoring the relevance of comprehensive antigen identification and prioritisation [18, 19]. At the same time, these findings emphasise the need for improved computational approaches capable of integrating diverse antigen sources and immunological features to support downstream experimental investigation.

Despite multiple efforts to develop antigen-directed strategies in GBM, including personalized neoantigen vaccines and multi-peptide vaccine approaches, durable clinical benefit has remained limited. Prior studies have primarily focused on mutation-derived neoantigens or predefined antigen panels, often without systematic comparison between mutation-derived TSAs, overexpression-based TAAs, and fusion-derived candidates within the same cohort. Consequently, the relative contribution, recurrence patterns, and predicted presentation characteristics of distinct antigen sources in GBM remain incompletely defined.

In this study, we performed whole-transcriptome sequencing of RNA extracted from 79 formalin-fixed paraffin-embedded (FFPE) GBM samples to characterize their tumour antigen landscape. We focused on transcriptionally expressed alterations, as mutant allele expression is a prerequisite for peptide generation and HLA presentation. We developed a multi-step analytical framework integrating mutation detection, HLA typing, gene expression analysis, population allele frequency filtering, MHC binding prediction, and antigen prioritization.

To our knowledge, this represents the first large-scale study to systematically analyse and directly compare mutation-derived TSAs, overexpression-derived TAAs, and fusion-derived candidates within a unified transcriptome-based framework in a clinically homogeneous GBM cohort. The resulting prioritized antigen landscape is intended to serve as a resource for downstream experimental validation and to inform rational antigen selection strategies in GBM immunotherapy research.

## Materials and methods

### RNA extraction, library preparation and sequencing

For this retrospective study conducted between 2023 and 2025, we obtained archived FFPE tumour samples collected between 2019 and 2025 from the Institute of Pathology, University of Ljubljana, Slovenia. All samples had a confirmed histopathological diagnosis of glioblastoma and were obtained at initial diagnosis prior to treatment. Tumour purity was assessed by a neuropathologist on haematoxylin and eosin stained sections. We extracted total RNA from tumour samples using the Maxwell RSC RNA FFPE Kit (Promega Corporation, Madison, WI, USA). Prior to library preparation, we removed genomic DNA (DNA Removal Add-on) and depleted ribosomal RNA (RiboCop HMR V2 rRNA Depletion Kit (Lexogen GmbH, Vienna, Austria). RNA inputs ranged from 70 to 1000 ng and were within the manufacturer’s recommended range for the Corall FFPE RNA-Seq Library Preparation Kit (Lexogen GmbH, Vienna, Austria). cDNA libraries were constructed according to the manufacturer’s protocol. In accordance with the Corall FFPE workflow, the number of PCR amplification cycles was empirically determined for each sample using the initial amplification step to optimize cycle number based on input quantity and amplification performance. Across the cohort, PCR cycles ranged from 11 to 15 cycles (median 12). Libraries were sequenced on the Illumina NovaSeq platform, and standard quality control and equimolar pooling procedures were performed prior to sequencing. Sequencing generated at least 30 million reads per sample, with total read counts ranging from 34.9 to 60.1 million reads.

In this study, only RNA was extracted from archived FFPE tumour tissue. Whole-exome sequencing was not performed due to limited available material and the retrospective nature of the cohort. Our analytical framework therefore focused on expressed variants detectable at the transcript level, enabling integration of mutation detection, expression quantification, and antigen prioritization within a single dataset.

### HLA typing, mutation detection

We performed HLA class I typing (HLA-A, HLA-B and HLA-C) directly from the RNA-seq FASTQ input using OptiType [20]. For somatic variant calling, we analysed the FASTQ files using the CTAT-Mutation pipeline (https://github.com/NCIP/ctat-mutations), which aligns reads to the GRCh38 reference genome using STAR Aligner [21]. After the initial call, we applied stringent filters to the resulting Variant Call Format (VCF) files and selected only variants with a Phred quality score of ≥ 30, ≥ 10 supporting reads and ≥ 3 reads supporting the mutant allele. We then annotated these filtered variants using Ensembl Variant Effect Predictor (VEP) [22]and processed them with the vt tool (https://genome.sph.umich.edu/wiki/Vt) to split multiallelic records. Prioritized variants were additionally annotated with population allele frequencies from gnomAD (GRCh38) [23], and variants with maximum allele frequency ≥ 0.001 were excluded from the final candidate set. We quantified gene expression using Kallisto [24], obtained variant-specific read counts using bam-readcount[25], and incorporated this information with the vcf-expression-annotator from VAtools (https://github.com/griffithlab/VAtools). To assess the potential influence of FFPE-related artefacts, we also analysed the nucleotide substitution spectrum of all single nucleotide variants. For this purpose, we normalised the substitutions to the six canonical classes (C > A, C > G, C > T, T > A, T > C, T > G) and summarised the counts. No matched normal tissue (blood, buccal, or adjacent non-diseased tissue) was available for this retrospective cohort.

### Tumour specific antigen prediction from SNVs

We identified TSA candidates derived from SNVs using the pVACseq [26] module from the pVACtools suite (v1.5.3) [26]. As part of this workflow, we generated 8–10-mer peptides from variants that were prefiltered based on gene expression levels and sequencing coverage. For each generated peptide, HLA binding affinity was predicted using NetMHCpan [27] and MHCflurry [28], proteasomal cleavage using NetChop [29], peptide MHC stability using NetMHCpan_stab [30], matches with reference protein sequence using Peptide Match (https://proteininformationresource.org/download/peptide_match/) and immunogenicity using the IEDB analysis resource [31].

### TAA candidate gene selection and peptide prediction

Our list of TAAs was compiled by integrating several analytical approaches. We first performed differential gene expression (DGE) analysis using DESeq2 [32], comparing our 79 GBM samples with normal brain cortex data from the GTEx portal. We considered genes with a log2 fold-change > 2 and an adjusted *p*-value < 0.01 as significantly overexpressed. Because all tumours were FFPE and all normal samples were GTEx (fresh-frozen), the batch and condition were perfectly confounded; we therefore modelled the condition only and used batch adjustment solely for visualization to illustrate potential technical effects. Second, we narrowed the selection by including curated genes known for their oncogenic roles (OncoKB) [33], those defined as cancer-testis antigens (CTDatabase, http://www.cta.lncc.br/index.html), and genes previously described as being robustly expressed in GBM [34]. From this selection, we retained in our cohort only genes with a baseline expression z-value > 1. This combined strategy resulted in a final list of 71 TAA source genes. We then obtained their protein sequences from ENSEMBL BioMart [35] and used the pVACbind module to predict the TAA peptide candidates.

### Fusion detection, validation and immunogenicity

To identify fusion transcripts, we used a consensus approach with three separate fusion callers: STAR-Fusion [36], Arriba [37] and FusionCatcher [38]. We retained only high-probability fusions that were recognised by at least two of these algorithms and supported by more than five junction reads. For this filtered set, we performed functional annotation using AGFusion (https://github.com/murphycj/agfusionweb-react/) and then predicted potential fusion-derived neoantigens with pVACfuse.

We experimentally validated the predicted fusions using Sanger sequencing. We first designed fusion-specific primers using the PrimerQuest tool (Integrated DNA Technologies) and amplified the target regions by PCR using FastGene Optima HotStart ReadyMix (Nippon Genetics Europe). We confirmed the presence of amplicons by visualising the PCR products on a 2% agarose gel and purified them using ExoSAP-IT (Applied Biosystems). After purification, we prepared the sequencing reaction by combining 2 µL of the product with 1 µL of a forward or reverse primer using BigDye Terminator Cycle Sequencing Kit 3.1 (Thermo Fisher Scientific). After a final purification step with BigDye XTerminator (Thermo Fisher Scientific), we sequenced the products on a SeqStudio platform, and analysed the resulting data with SeqScanner software and Ensembl Genome Browser.

### Final filtering and prioritisation of candidates

**Fig. 1:**
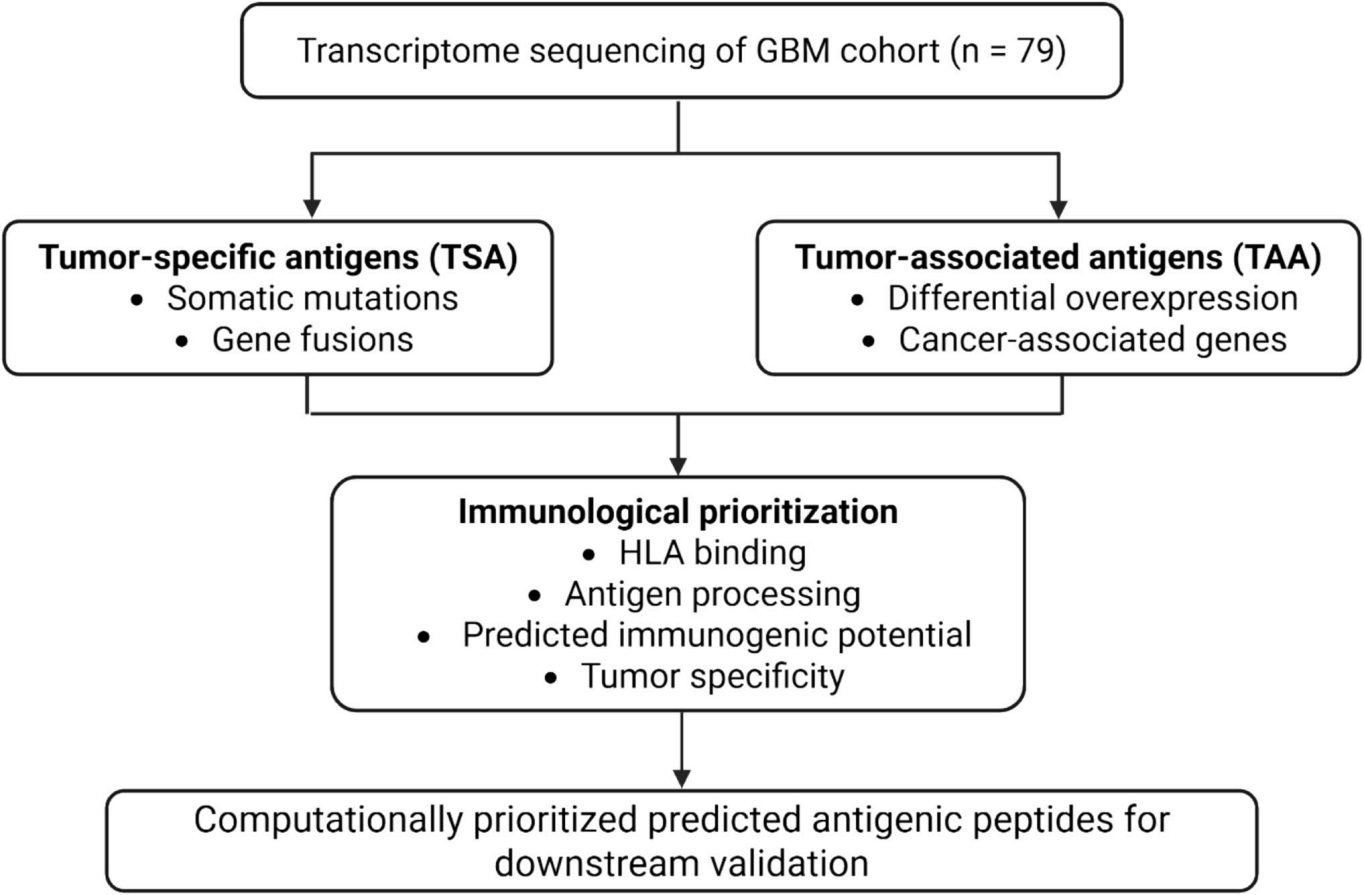
Overview of transcriptome-based identification of TSAs and TAAs in GBM. Transcriptome sequencing data from 79 GBM samples were used to identify mutation- and fusion-derived TSA as well as expression-derived TAA, which were subsequently prioritized based on predicted immunological features for downstream validation. Created with Biorender.com

Finally, we applied a uniform set of stringent criteria to prioritise the candidates from all three approaches. We retained a peptide as a strong candidate only if it had a predicted binding affinity < 500 nM, cleavage score > 0.5, predicted stability score > 0.5, and IEDB immunogenicity score > 0. For SNV-derived TSAs, we applied the following requirements: a tumour RNA variant allele frequency (VAF) > 0.1, sequencing depth ≥ 10, and no matches with the reference proteome, as confirmed by the peptide match tool (Fig. 1).

### Functional enrichment analysis

Functional enrichment analysis of recurrent TAA source genes was performed using the Enrichr [39] platform. Gene sets were queried against KEGG, Reactome, and Gene Ontology (Biological Process) databases. Pathways were ranked by adjusted p-value, and the top enriched terms were retained for reporting.

### Comparison with previously reported GBM antigen studies

Prioritized antigen candidates were compared at gene and peptide levels with previously published GBM vaccine and immunopeptidomics studies. Overlap was assessed manually based on reported gene symbols and peptide sequences.

Exact peptide sequence matching was performed against the IEDB MHC ligand dataset (mhc_ligand_full). Records were filtered to Homo sapiens, MHC class I, and positive ligand assays. Matches were assessed at the peptide level and, where available, at the peptide plus HLA allele level.

### Immune cell estimation and correlation analysis

Immune cell populations were estimated from transcriptome data using MCP-counter [40]. Spearman correlation analysis was performed to assess associations between the number of prioritized TSA and TAA candidates per sample and inferred immune cell signatures. P-values were adjusted using the Benjamini–Hochberg method.

## Results

For our analysis we selected 79 FFPE GBM, IDH-wild type, CNS WHO grade 4 tumour samples. Our cohort comprised 52 males and 27 females, with a mean age of 62.1 years. A summary of key demographic characteristics can be found in Table 1. All libraries met sequencing quality thresholds, with a minimum of 34.8 million reads per sample (range 34.8–60.1 million reads).

**Table 1:** Demographic data of the analysed cohort

Characteristic	Value
Age	62.1 ± 11.3 (range 30–82)
Sex	M: 52 F: 27
MGMT methylation	Unmethylated: 52 Methylated: 25 Not performed: 2
Tumour purity (%)	81.39 ± 8.98 (range 40–90)

### Antigen prioritization metrics

To ensure the quality of the variant calls from FFPE samples, we first applied a stringent technical filter to minimise fixation-related artefacts. Specifically, we restricted our analysis to variants supported by tumour RNA coverage of at least 10 reads and a minimum of three alternate reads (range 3–341). The median tumour RNA VAF was 0.33 (range 0.25–1.0). Although RNA-based VAF is influenced by transcript abundance and does not directly reflect cancer cell fraction, the observed distribution suggests that many prioritized variants were present in a substantial fraction of tumour cells. Definitive clonality assessment would require tumour–normal DNA sequencing with copy number integration. Variants with population allele frequency ≥ 0.001 in gnomAD were excluded prior to antigen prioritization. Following initial technical filtering, remaining candidates were prioritised using computational immunological features reported in the literature.

Prioritisation criteria included predicted peptide-HLA binding affinity (IC50), proteasomal cleavage score, peptide-HLA complex stability and predicted immunogenic potential. Application of an IC50 threshold resulted in the removal of approximately 92.2% (SD ± 2.3%) of the TSA candidates and 99.99% of the TAA candidates predicted by pVACseq or pVACbind. A subsequent multi-parameter filtering step required candidates to meet a cleavage score > 0.5, stability score > 0.5 and positive predicted immunogenicity score (IEDB > 0). Notably, in one sample (GB_27), no TSA candidates satisfied all combined criteria. For prioritized TSA variants, the median tumour RNA depth at the variant locus was 16 reads (range 10–841).

After completing this prioritisation cascade, we identified a mean of 21.3 TSA candidates per sample (SD ± 16.8, range 0–61). For the TAA candidates, derived from a curated list of 71 genes overexpressed in GBM relative to normal brain cortex (Supplementary Table 2), the same filtering process yielded a mean of 46.6 peptides per sample (SD ± 15.2, range 8–73).

### Fusion-derived antigen candidates

We detected gene fusions in 26 of our 79 samples (32.9%), with 46 distinct fusion events identified across the cohort, with six samples harbouring multiple fusions. Of these, 36 were predicted to encode protein-coding transcripts and were validated by Sanger sequencing, and 16 of these were subsequently predicted to generate candidate peptides with potential for HLA presentation. However, after applying our stringent prioritisation criteria, our analysis revealed four fusion-derived peptide candidates in three samples. A comprehensive overview of all detected fusions is provided in Supplementary Table 3.

### HLA presentation of predicted antigen candidates

To assess the influence of HLA genotype on antigen presentation, we analysed class I HLA profiles across the cohort, and identified 1633 TSA candidates and 544 TAA candidates. Overall, we observed 90 different HLA alleles across the HLA-A, -B and -C loci (Supplementary Figs. 1 and 2). We confirmed that the allele frequencies in our cohort were consistent with those reported in the Slovene population in the Allele Frequency Net Database [41]. The most frequent HLA-A alleles were A*02:01 (20.3%), A*01:01 (19.0%) and A*03:01 (12.7%). At the HLA-B locus, B*07:02 (14.6%) and B*18:01 (7.6%) were the most common alleles, whereas C*07:02 (20.9%) was the most common allele at the HLA-C locus.

Despite this diversity, predicted peptide presentation was disproportionately associated with a limited subset of alleles. We observed that the highest number of candidate peptides was restricted to HLA-A alleles: A*02:01 (present in 29 samples; restriction of 550 TSA and 61 TAA peptides), A*01:01 (29 samples; 39 TSA and 16 TAA peptides), and A*03:01 (19 samples; 154 TSA and 34 TAA peptides). At the HLA-B locus, presentation was primarily associated with B*07:02, B*18:01 and B*35:01. In contrast, predicted presentation via HLA-C alleles was limited, with only C*02:02 contributing to TSA candidates and C*02:02 and C*12:02 contributing to TAA candidates.

When we quantified these contributions by locus, we confirmed that HLA-A alleles accounted for the majority of predicted peptide presentation (74.3% of TSAs and 67.1% of TAAs), followed by HLA-B (25.2% of TSAs and 32.2% of TAAs). HLA-C alleles contributed only a minor fraction of predicted peptide presentation (0.5% of TSAs and 0.7% of TAAs). This skewed distribution suggests that antigen discovery efforts may disproportionately favour common HLA-A alleles, potentially limiting coverage in less frequent genotypes.

### Comparative analysis of TSA and TAA landscape

**Fig. 2:**
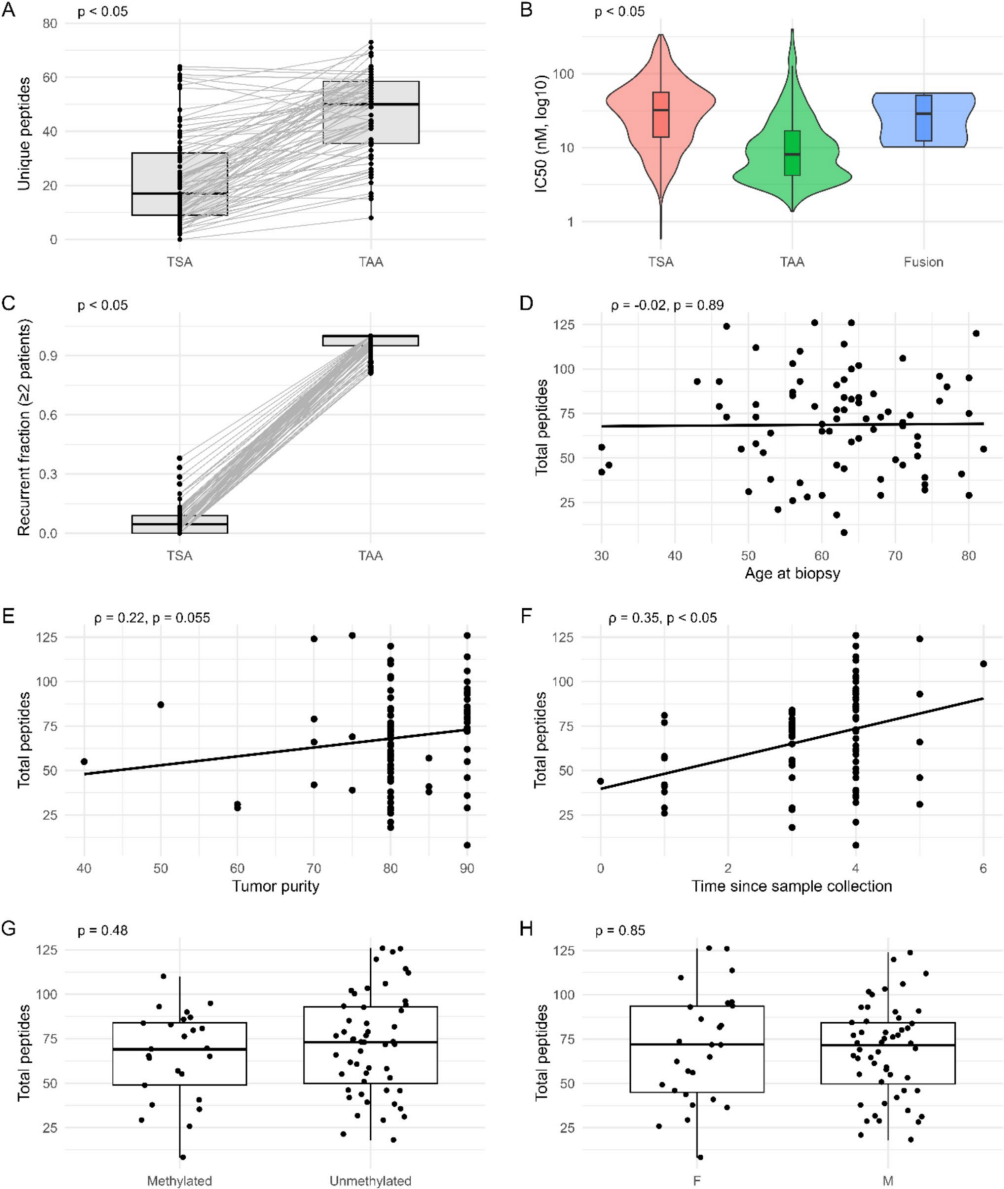
Statistical comparison of TSA and TAA landscapes across the GBM cohort. Fusion-derived peptides were excluded from these analyses due to their low frequency. **A** paired comparison of predicted peptide burden per sample, showing a significantly higher number of TAA candidates compared with TSA candidates (paired Wilcoxon signed-rank test). **B** distribution of predicted peptide–HLA binding affinities (IC50, nM; log_10_ scale) for TSA and TAA candidates, showing differences in predicted IC50 values between groups (Kruskal–Wallis test). **C** paired comparison of recurrent peptides (defined as peptides detected in ≥ 2 patients), demonstrating higher recurrence among TAAs (paired Wilcoxon signed-rank test). **D**–**F** associations between total predicted peptide burden and patient age at biopsy (**D**), tumour purity (**E**), and time since sample collection (**F**), assessed using Spearman’s rank correlation. **G**, **H** comparison of total predicted peptide burden by MGMT promoter methylation status (**G**) and patient sex (**H**), assessed using unpaired Wilcoxon rank-sum tests

**Fig. 3:**
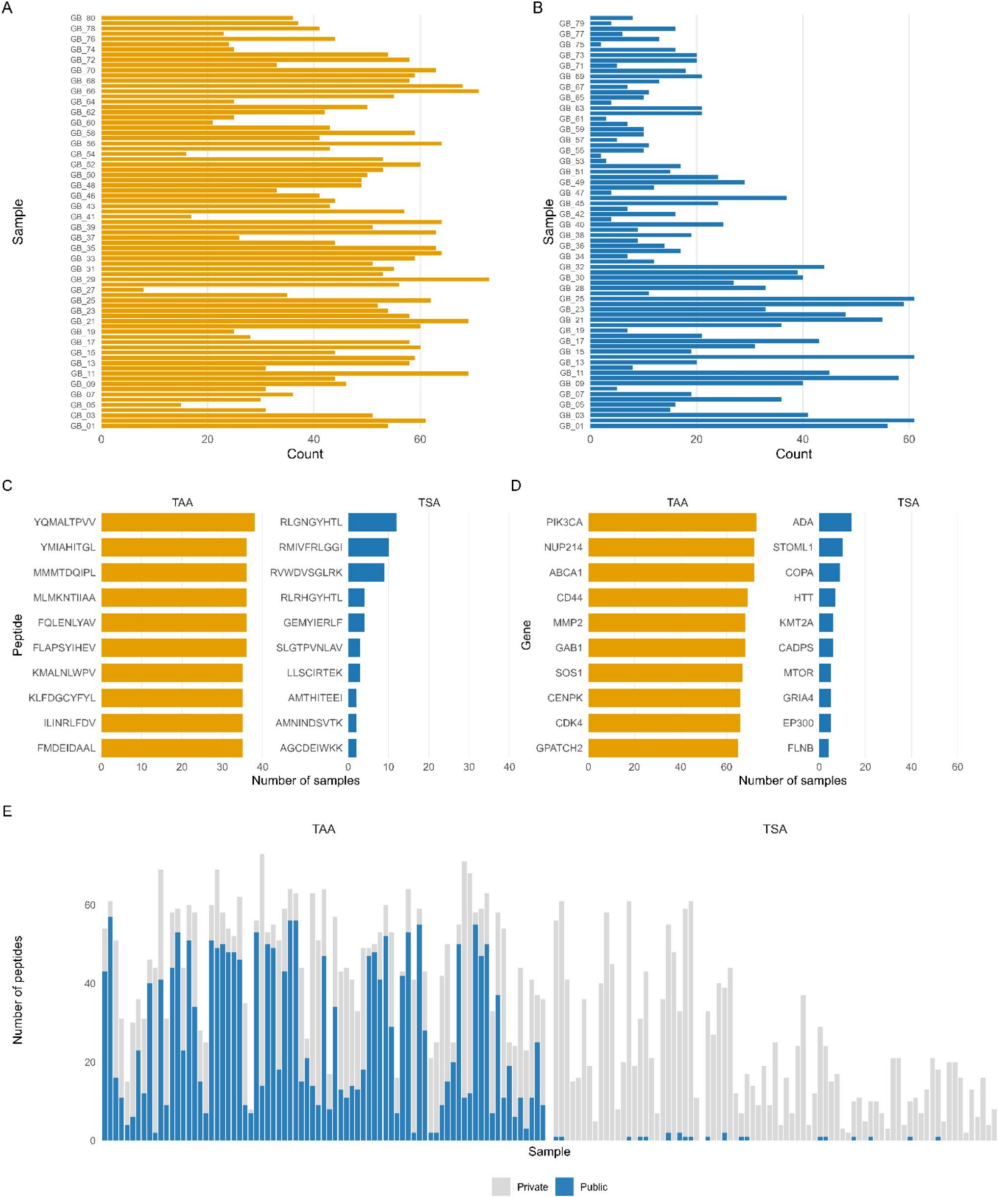
Distribution and recurrence of prioritized predicted TSAs and TAAs across the GBM cohort. **A**, **B** number of prioritized peptides per sample for TAA (**A**) and TSA (**B**). **C** most recurrent prioritized peptides and **D** genes contributing recurrent peptides within each group. **E** distribution of prioritized peptides across samples, distinguishing shared (“public”) and sample-specific (“private”) candidates. All peptides shown represent in silico predictions prioritized for downstream validation

We next performed a statistical comparison of the TSA and TAA candidate landscape, focusing on peptide burden, recurrence and binding strength (Fig. 2). The final distribution of predicted TAA and TSA candidate peptides per sample following all filtering steps is shown in Fig. 3A, B respectively.

Our analysis revealed that the TAA group had a significantly higher predicted peptide burden per sample than the TSA group (paired Wilcoxon test, *p* = 9.6 × 10^−14^). Similarly, we found that the proportion of recurrent peptides (defined as peptides detected in two or more patients) was significantly greater among the TAAs (Wilcoxon test, *p* = 1.1 × 10^−14^). When assessing the predicted binding affinity, our analysis showed that the TAA candidates demonstrated significantly lower IC50 values and had lower IC50 values than the TSA candidates (Kruskal–Wallis, *p* < 2.2 × 10^−16^).

We further assessed the relationship between total predicted peptide burden and clinical or sample characteristics. No significant associations were observed with patient age at biopsy (R = − 0.02, *p* = 0.89), MGMT promoter methylation status (Wilcoxon test, *p* = 0.48), or patient sex (Wilcoxon test, *p* = 0.85). A weak positive trend was observed between peptide burden and tumour purity, although this did not reach statistical significance (R = 0.22, *p* = 0.055). In contrast, a moderate positive correlation was observed between peptide burden and time since sample collection (R = 0.35, *p* = 0.0018).

To explore whether predicted antigen burden was associated with immune infiltration, we estimated immune cell populations using MCP-counter applied to transcriptome data. Spearman correlation analysis revealed no statistically significant associations between TSA or TAA burden and major immune compartments after correction for multiple testing (Benjamini–Hochberg false discovery rate > 0.05 for all comparisons; Supplementary Table 5). A nominal positive association between TSA burden and NK cell signature scores was observed (ρ = 0.30, raw *p* = 0.008), but this did not remain significant following correction.

### Shared versus private candidate peptides

We next examined peptide recurrence to distinguish sample-specific from shared antigen candidates. As expected, TSA candidates were predominantly private, with only 22 peptides (1.3%) shared between two or more samples. In contrast, 431 TAA candidates (11.7%) were detected in at least two samples (Fig. 3E). This difference was statistically significant, since the median proportion of shared peptides per sample was much higher for TAAs than for TSAs (paired Wilcoxon test, *p* = 1.1 × 10^−14^).

Although TSA recurrence was generally limited, several peptides were observed in multiple samples. The peptide RLGNGYHTL was identified in 12 samples and resulted from recurrent ADA mutations (c.712C > A, p.His238Asn; and in one case c.640C > A, p.His214Asn). Another TSA candidate, RMIVFRLGGI, was detected in 10 samples due to a recurrent STOML1 mutation (c.283C > G, p.Arg95Gly) (Fig. 3C).

In contrast, the TAA candidates showed broad recurrence across the cohort. The most abundant TAA peptide, YQMALTPVV, derived from CDK4, was present in 38 samples (48%). Additional recurrent peptides originated from genes such as ATAD2, MTHFD2, and TYMS, each present in more than 30 samples. At the gene level, predicted TAAs from PIK3CA, NUP214, and ABCA1 were detected in over 72 samples (Fig. 3D).

Analysis of HLA restriction indicated that recurrent peptides were predominantly associated with HLA-A alleles, followed by HLA-B, with minimal contribution from HLA-C. The strongest recurrence was observed for peptides restricted by A*02:01, A*03:01, B*07:02, and B*35:01, suggesting that specific HLA backgrounds may favour presentation of shared TAA candidates.

To contextualise these findings with respect to potential on-target and off-tumour expression, we examined the expression of the 71 TAA source genes across more than 50 normal tissues using GTEx v10 data. Most candidate genes showed minimal expression in vital organs (Supplementary Fig. 3). Several cancer-testis antigens, including *MAGEB3* and *PBK*, exhibited negligible expression across normal tissues, whereas other genes such as *CDK4* and *PIK3CA* showed measurable expression in tissues including liver and brain. A subset of candidates, including *CTCFL, OIP5,* and *TEX101*, displayed expression restricted to immune-privileged tissues, consistent with their classification as cancer-testis antigens. This integrative analysis supports prioritisation of TAA candidates based on recurrence, predicted presentation, and normal tissue expression profiles.

### Comparison with previously reported GBM antigen targets

To contextualize our prioritized antigen candidates within existing GBM immunotherapy research, we compared our gene and peptide lists with previously reported GBM vaccine and immunopeptidomics studies, including Dutoit et al. [42], GAPVAC/APVAC [43–45] and Latzer et al. [18] (Supplementary Table 6). At the gene level, multiple overlaps were identified across both antigen classes. For example, *TOP2A* (Dutoit et al.) and *CDK4* (GAPVAC/APVAC1) were present among our prioritized TAA candidates. Among mutation-derived TSAs, overlapping genes included *PTEN, TP53, EP300, ARHGAP35*, and others reported in personalized vaccine studies. Peptide-level concordance was limited, with one exact *EGFR*-derived peptide match identified in the Latzer et al. cohort. The limited peptide overlap likely reflects inter-patient heterogeneity in mutation landscapes.

To assess whether predicted candidates have prior experimental support for HLA presentation, we performed exact sequence matching against the IEDB human MHC class I ligand dataset (positive ligand assays). Of 2,182 unique prioritized peptides, 115 (5.3%) had an exact match in the IEDB ligandome, and 56 (2.6%) additionally matched the same restricting HLA allele reported in IEDB (Supplementary Table 7). These findings provide orthogonal evidence that a subset of predicted candidates have previously been detected as HLA class I ligands.

### Functional enrichment of recurrent TAA source genes

To investigate whether recurrent TAA candidates reflected shared biological programs, we performed pathway enrichment analysis of TAA source genes detected in at least two patients. Reactome analysis revealed significant enrichment of cell cycle–related pathways, including Cell Cycle, Mitotic Cell Cycle, and Cell Cycle Checkpoints. KEGG pathway analysis demonstrated enrichment in PI3K–AKT signaling and Proteoglycans in cancer pathways. Gene Ontology analysis further highlighted processes related to positive regulation of transcription, cell population proliferation, and mitotic chromosome alignment (Supplementary Table 4). In contrast to TAA candidates, TSA-derived genes did not demonstrate clear pathway convergence, consistent with the predominantly private and heterogeneous nature of mutation-derived epitopes in GBM.

## Discussion

To our knowledge, this study provides one of the first systematic side-by-side evaluations of mutation-derived TSAs, overexpression-derived TAAs, and fusion-derived candidates within a unified transcriptome-based framework in a clinically homogeneous IDH-wildtype GBM cohort. Rather than introducing a novel prediction algorithm, our objective was to comparatively characterize distinct antigen sources under identical prioritization criteria and to generate a rigorously curated catalogue of transcriptionally expressed candidates for downstream validation.

### Comparative landscape of TSAs and TAAs in GBM

To generate this dataset, we developed a transcriptome-based antigen prioritisation workflow compatible with archival FFPE specimens, enabling systematic evaluation of expressed mutations and expression-derived candidates in a retrospective clinical cohort [46, 47]. Although the use of FFPE tissue presents technical challenges, such as fixation artefacts, we were able to mitigate these through stringent filtering criteria and exclusion of variants with low read support [48]. Initial prioritisation using a conservative binding affinity threshold (IC50 < 500 nM) substantially reduced the candidate space, and subsequent filtering based on cleavage probability, peptide–MHC stability, and predicted immunogenic potential yielded a refined set of high-confidence candidate peptides. Using transcriptome sequencing, we focussed exclusively on expressed mutations and filtered out variants that were not transcribed [46].

Mutation-derived TSAs were predominantly private to individual tumours, reflecting the relatively low mutational burden and inter-patient heterogeneity characteristic of GBM. Although many prioritized TSAs demonstrated robust RNA support and substantial alternate allele representation, they rarely recurred across patients. This private nature is consistent with prior neoantigen vaccine studies in GBM and reinforces the logistical complexity of broadly applicable mutation-based vaccination strategies in this disease context.

In contrast, TAA-derived candidates formed a larger and more recurrent antigen pool. Recurrent TAAs were frequently derived from genes central to proliferative and oncogenic programs, including *CDK4*, *MTHFD2*, and *PIK3CA*. Pathway enrichment analysis further demonstrated convergence on cell cycle and growth signalling pathways, suggesting that recurrent TAAs reflect shared biological programs rather than random overexpression events. Because such genes often play essential roles in tumour maintenance, epitopes derived from them may be less susceptible to clonal antigen loss compared with TSAs.

Together, these findings support a complementary antigen model in GBM. Personalized TSAs offer high tumour specificity but limited shared applicability, whereas recurrent TAAs may enable shared or semi-personalized targeting strategies. A rational immunotherapeutic framework may therefore benefit from integrating both antigen classes, balancing tumour specificity with recurrence and population level feasibility.

### Gene fusions as a niche antigen source

Consistent with previous studies, gene fusions represented a rare source of candidate tumour antigens in GBM. Nevertheless, they remain of interest because they arise exclusively in tumour cells and may generate structurally distinct epitopes, particularly in cases involving frameshift events. In our cohort, fusions were infrequent and often localised to chromosomal regions previously implicated in GBM chromothripsis, such as chromosomes 7 and 12 [49]. Although only a handful of fusion peptides satisfied our stringent filtering, their absolute tumour specificity supports their consideration as a distinct antigen class warranting further investigation.

### Influence of HLA genotype

Our analysis confirmed that the HLA genotype is a key determinant of predicted antigen presentation. Although a broad range of HLA-A, -B, and -C alleles was observed across the cohort, predicted peptide presentation was disproportionately associated with a limited subset of alleles, most notably HLA-A*02:01, A*01:01, A*03:01, and B*07:02. This distribution is consistent with allele frequencies reported for the Slovene population, supporting the representativeness of the cohort. In contrast, HLA-C alleles contributed only minimally to the predicted antigen pool, likely reflecting their more restrictive peptide-binding repertoires. Notably, allele frequency did not necessarily correspond to predicted antigen presentation capacity, as illustrated by the limited contribution of C*07:02 despite its high prevalence. This skewed distribution highlights an important translational consideration that antigen discovery and vaccine design strategies may inherently favour common HLA-A genotypes. Consideration of population-level HLA distribution will therefore be essential for ensuring equitable applicability of antigen-targeted therapies.

### Biological and translational implications

Exploratory immune deconvolution analysis demonstrated no significant association between predicted antigen burden and estimated immune infiltration after correction for multiple testing. Although a nominal correlation between TSA burden and NK cell signature was observed, it did not remain significant after FDR adjustment. These findings are consistent with the immunosuppressive microenvironment characteristic of GBM and suggest that antigen availability alone is insufficient to predict immune engagement.

Collectively, our results indicate that multiple antigen classes contribute to the GBM antigen landscape but differ substantially in recurrence and predicted stability. The predominance of private TSAs underscores the challenges of broadly applicable neoantigen-based vaccines in GBM, whereas recurrent TAA-derived candidates may support complementary shared or semi-personalized targeting approaches. These data reinforce the need for integrated strategies combining rational antigen selection with modulation of tumour microenvironment barriers.

### Context with prior GBM antigen and vaccine studies

Our findings are broadly consistent with prior GBM antigen-directed efforts, which have reported limited numbers of mutation-derived neoantigens per patient and marked inter-patient heterogeneity. When we compared our prioritized candidates with previously published GBM vaccine and immunopeptidomics studies (including Dutoit et al., Keskin et al., Johanns et al., and Latzer et al.), we observed multiple gene-level overlaps across both antigen classes (e.g., *CDK4, PTEN, TP53, EP300*), while peptide-level concordance was rare. The limited peptide overlap likely reflects differences in individual tumour mutation landscapes, cohort HLA distributions, and the dependence of predicted peptide generation on allele-specific processing and presentation.

To further contextualize our predictions, we performed peptide-level comparison against the IEDB MHC class I ligand dataset. A subset of predicted candidates demonstrated exact sequence matches to previously reported human HLA ligands, including cases with allele-concordant restriction. These findings provide independent orthogonal support that selected peptides derived from our workflow are detectable within experimentally defined immunopeptidomes. Importantly, the majority of predicted peptides did not have prior IEDB matches, which is expected given the limited coverage of tumour immunopeptidomics datasets, HLA allele bias in existing studies, and context-specific nature of antigen presentation. Thus, absence of prior documentation does not preclude biological presentation but underscores the need for tumour-specific validation.

Notably, Latzer et al. reported real-world application of personalized peptide vaccination in GBM, demonstrating induction of neoantigen-specific immune responses but limited durable clinical control. Their findings underscore both the feasibility and the biological constraints of neoantigen-targeted strategies in GBM, particularly the challenge of selecting robust and clinically meaningful targets within a highly heterogeneous tumour landscape. Our observation that TSAs are predominantly private, whereas TAAs display greater recurrence across patients, provides a complementary perspective on antigen selection and supports further evaluation of integrated multi-antigen strategies combining recurrent TAAs with personalized TSAs.

### Methodological considerations and limitations

Several methodological considerations and limitations should be considered. First, antigen candidates were identified using computational prediction of antigen processing, HLA binding, and immunogenicity. Although multi-parameter filtering was applied to enrich for biologically plausible candidates, in silico prediction does not guarantee in vivo peptide presentation or T-cell recognition, and experimental validation will be required.

Second, the absence of matched tumour–normal DNA limits definitive somatic classification. While stringent technical thresholds and population allele frequency filtering were applied to reduce inclusion of common germline variants, rare germline alterations or low-level mosaicism cannot be fully excluded without paired normal sequencing. Mutation-derived candidates should therefore be considered prioritized and putative pending orthogonal DNA-based validation.

Third, RNA-based variant detection is influenced by transcript abundance and non-uniform coverage, prioritizing expressed alterations while potentially under-detecting lowly expressed or non-transcribed variants. RNA-derived VAF cannot be equated with cancer cell fraction, and definitive clonality assessment would require tumour–normal DNA sequencing with copy number integration.

Finally, immune infiltration was inferred using transcriptomic deconvolution rather than spatial or histopathological methods, and thus does not capture functional or spatial immune heterogeneity within the tumour microenvironment.

Despite these limitations, the present study provides a systematically prioritized and comparatively evaluated resource of transcriptionally expressed GBM antigen candidates intended to support downstream experimental validation and translational research.

## Supplementary Information

Below is the link to the electronic supplementary material.

Supplementary file1 (DOCX 2532 KB)

## Data Availability

The data presented in this study are available on request from the corresponding author. The normal tissue dataset analysed in this study was obtained from: [https://www.gtexportal.org/home/downloads/adult-gtex/bulk_tissue_expression#bulk_tissue_expression-gtex_analysis_v10-rna-seq](https:/www.gtexportal.org/home/downloads/adult-gtex/bulk_tissue_expression), the Genotype-Tissue Expression (GTEx) Portal on 08/25/2025. The GTEx Project was supported by the Common Fund of the Office of the Director of the National Institutes of Health, and by NCI, NHGRI, NHLBI, NIDA, NIMH, and NINDS.

## References

[1] Schaff LR, Mellinghoff IK (2023) Glioblastoma and other primary brain malignancies in adults: a review. JAMA 329(7):574–587. 10.1001/jama.2023.0023 36809318 10.1001/jama.2023.0023 PMC11445779

[2] WHO Classification of Tumours Editorial Board (2021) World health organization classification of tumours of the central nervous system, 5th ed. International Agency for Research on Cancer, Lyon

[3] Lang F, Schrors B, Lower M, Tureci O, Sahin U (2022) Identification of neoantigens for individualized therapeutic cancer vaccines. Nat Rev Drug Discov 21(4):261–282. 10.1038/s41573-021-00387-y 35105974 10.1038/s41573-021-00387-y PMC7612664

[4] Verdugo E, Puerto I, Medina MA (2022) An update on the molecular biology of glioblastoma, with clinical implications and progress in its treatment. Cancer Commun (Lond) 42(11):1083–1111. 10.1002/cac2.12361 36129048 10.1002/cac2.12361 PMC9648390

[5] Salahlou R, Farajnia S, Alizadeh E, Dastmalchi S (2025) Recent developments in peptide vaccines against glioblastoma, a review and update. Mol Brain 18(1):50. 10.1186/s13041-025-01221-x 40514725 10.1186/s13041-025-01221-x PMC12166567

[6] Seager RJ, Senosain MF, Van Roey E, Gao S, DePietro P, Nesline MK et al (2024) Cancer testis antigen burden (CTAB): a novel biomarker of tumor-associated antigens in lung cancer. J Transl Med 22(1):141. 10.1186/s12967-024-04918-0 38326843 10.1186/s12967-024-04918-0 PMC10851610

[7] Rapp C, Warta R, Stamova S, Nowrouzi A, Geisenberger C, Gal Z et al (2017) Identification of T cell target antigens in glioblastoma stem-like cells using an integrated proteomics-based approach in patient specimens. Acta Neuropathol 134(2):297–316. 10.1007/s00401-017-1702-1 28332095 10.1007/s00401-017-1702-1

[8] Singh P, Khatib MN, Roopashree R, Kaur M, Srivastava M, Barwal A et al (2025) Advancements and challenges in personalized neoantigen-based cancer vaccines. Oncol Rev 19:1541326. 10.3389/or.2025.1541326 40160263 10.3389/or.2025.1541326 PMC11949952

[9] Katsikis PD, Ishii KJ, Schliehe C (2024) Challenges in developing personalized neoantigen cancer vaccines. Nat Rev Immunol 24(3):213–227. 10.1038/s41577-023-00937-y 37783860 10.1038/s41577-023-00937-y PMC12001822

[10] Liu Y, Zhou F, Ali H, Lathia JD, Chen P (2024) Immunotherapy for glioblastoma: current state, challenges, and future perspectives. Cell Mol Immunol 21(12):1354–1375. 10.1038/s41423-024-01226-x 39406966 10.1038/s41423-024-01226-x PMC11607068

[11] Lybaert L, Lefever S, Fant B, Smits E, De Geest B, Breckpot K et al (2023) Challenges in neoantigen-directed therapeutics. Cancer Cell 41(1):15–40. 10.1016/j.ccell.2022.10.013 36368320 10.1016/j.ccell.2022.10.013

[12] Bausart M, Preat V, Malfanti A (2022) Immunotherapy for glioblastoma: the promise of combination strategies. J Exp Clin Cancer Res 41(1):35. 10.1186/s13046-022-02251-2 35078492 10.1186/s13046-022-02251-2 PMC8787896

[13] Bhardwaj JS, Paliwal S, Singhvi G, Taliyan R (2024) Immunological challenges and opportunities in glioblastoma multiforme: a comprehensive view from immune system lens. Life Sci 357:123089. 10.1016/j.lfs.2024.123089 39362586 10.1016/j.lfs.2024.123089

[14] Tang J, Karbhari N, Campian JL (2025) Therapeutic targets in Glioblastoma: molecular pathways, emerging strategies, and future directions. Cells. 10.3390/cells14070494 40214448 10.3390/cells14070494 PMC11988183

[15] Lu L, Zhan M, Li XY, Zhang H, Dauphars DJ, Jiang J et al (2022) Clinically approved combination immunotherapy: current status, limitations, and future perspective. Curr Res Immunol 3:118–127. 10.1016/j.crimmu.2022.05.003 35676925 10.1016/j.crimmu.2022.05.003 PMC9167882

[16] Mahajan S, Schmidt MHH, Schumann U (2023) The Glioma immune landscape: a double-edged sword for treatment regimens. Cancers (Basel). 10.3390/cancers15072024 37046685 10.3390/cancers15072024 PMC10093409

[17] Dolton G, Rius C, Wall A, Szomolay B, Bianchi V, Galloway SAE et al (2023) Targeting of multiple tumor-associated antigens by individual T cell receptors during successful cancer immunotherapy. Cell 186(16):3333–3349. 10.1016/j.cell.2023.06.02037490916 10.1016/j.cell.2023.06.020

[18] Latzer P, Zelba H, Battke F, Reinhardt A, Shao B, Bartsch O et al (2024) A real-world observation of patients with glioblastoma treated with a personalized peptide vaccine. Nat Commun 15(1):6870. 10.1038/s41467-024-51315-8 39127809 10.1038/s41467-024-51315-8 PMC11316744

[19] Biswas N, Chakrabarti S, Padul V, Jones LD, Ashili S (2023) Designing neoantigen cancer vaccines, trials, and outcomes. Front Immunol 14:1105420. 10.3389/fimmu.2023.1105420 36845151 10.3389/fimmu.2023.1105420 PMC9947792

[20] Szolek A, Schubert B, Mohr C, Sturm M, Feldhahn M, Kohlbacher O (2014) OptiType: precision HLA typing from next-generation sequencing data. Bioinformatics 30(23):3310–3316. 10.1093/bioinformatics/btu548 25143287 10.1093/bioinformatics/btu548 PMC4441069

[21] Dobin A, Davis CA, Schlesinger F, Drenkow J, Zaleski C, Jha S et al (2013) STAR: ultrafast universal RNA-seq aligner. Bioinformatics 29(1):15–21. 10.1093/bioinformatics/bts635 23104886 10.1093/bioinformatics/bts635 PMC3530905

[22] McLaren W, Gil L, Hunt SE, Riat HS, Ritchie GR, Thormann A et al (2016) The Ensembl variant effect predictor. Genome Biol 17(1):122. 10.1186/s13059-016-0974-4 27268795 10.1186/s13059-016-0974-4 PMC4893825

[23] Chen S, Francioli LC, Goodrich JK, Collins RL, Kanai M, Wang Q et al (2024) A genomic mutational constraint map using variation in 76,156 human genomes. Nature 625(7993):92–100. 10.1038/s41586-023-06045-0 38057664 10.1038/s41586-023-06045-0 PMC11629659

[24] Bray NL, Pimentel H, Melsted P, Pachter L (2016) Near-optimal probabilistic RNA-seq quantification. Nat Biotechnol 34(5):525–527. 10.1038/nbt.3519 27043002 10.1038/nbt.3519

[25] Khanna A, Larson D, Srivatsan S, Mosior M, Abbott T, Kiwala S et al (2022) Bam-readcount - rapid generation of basepair-resolution sequence metrics. J Open Source Softw. 10.21105/joss.03722 10.21105/joss.03722 PMC13367885 42453900

[26] Hundal J, Kiwala S, McMichael J, Miller CA, Xia H, Wollam AT et al (2020) pVACtools: a computational toolkit to identify and visualize cancer neoantigens. Cancer Immunol Res 8(3):409–420. 10.1158/2326-6066.CIR-19-0401 31907209 10.1158/2326-6066.CIR-19-0401 PMC7056579

[27] Reynisson B, Alvarez B, Paul S, Peters B, Nielsen M (2020) NetMHCpan-4.1 and NetMHCIIpan-4.0: improved predictions of MHC antigen presentation by concurrent motif deconvolution and integration of MS MHC eluted ligand data. Nucleic Acids Res 48(W1):W449–W454. 10.1093/nar/gkaa379 32406916 10.1093/nar/gkaa379 PMC7319546

[28] O’Donnell TJ, Rubinsteyn A, Laserson U (2020) MHCflurry 2.0: improved pan-allele prediction of MHC class I-presented peptides by incorporating antigen processing. Cell Syst 11(1):42–48. 10.1016/j.cels.2020.06.010 32711842 10.1016/j.cels.2020.06.010

[29] Nielsen M, Lundegaard C, Lund O, Kesmir C (2005) The role of the proteasome in generating cytotoxic T-cell epitopes: insights obtained from improved predictions of proteasomal cleavage. Immunogenetics 57(1–2):33–41. 10.1007/s00251-005-0781-7 15744535 10.1007/s00251-005-0781-7

[30] Rasmussen M, Fenoy E, Harndahl M, Kristensen AB, Nielsen IK, Nielsen M, Buus S (2016) Pan-specific prediction of peptide-MHC class I complex stability, a correlate of T cell immunogenicity. J Immunol 197(4):1517–1524. 10.4049/jimmunol.1600582 27402703 10.4049/jimmunol.1600582 PMC4976001

[31] Vita R, Blazeska N, Marrama D, Members Iedb Curation Team, Duesing S, Bennett J et al (2025) The immune epitope database (IEDB): 2024 update. Nucleic Acids Res 53(D1):D436–D443. 10.1093/nar/gkae1092 39558162 10.1093/nar/gkae1092 PMC11701597

[32] Love MI, Huber W, Anders S (2014) Moderated estimation of fold change and dispersion for RNA-seq data with DESeq2. Genome Biol 15(12):550. 10.1186/s13059-014-0550-8 25516281 10.1186/s13059-014-0550-8 PMC4302049

[33] Suehnholz SP, Nissan MH, Zhang H, Kundra R, Nandakumar S, Lu C et al (2024) Quantifying the expanding landscape of clinical actionability for patients with cancer. Cancer Discov 14(1):49–65. 10.1158/2159-8290.CD-23-0467 37849038 10.1158/2159-8290.CD-23-0467 PMC10784742

[34] Tang J, He D, Yang P, He J, Zhang Y (2018) Genome-wide expression profiling of glioblastoma using a large combined cohort. Sci Rep 8(1):15104. 10.1038/s41598-018-33323-z 30305647 10.1038/s41598-018-33323-z PMC6180049

[35] Dyer SC, Austine-Orimoloye O, Azov AG, Barba M, Barnes I, Barrera-Enriquez VP et al (2025) Ensembl 2025. Nucleic Acids Res 53(D1):D948–D957. 10.1093/nar/gkae1071 39656687 10.1093/nar/gkae1071 PMC11701638

[36] Haas BJ, Dobin A, Li B, Stransky N, Pochet N, Regev A (2019) Accuracy assessment of fusion transcript detection via read-mapping and de novo fusion transcript assembly-based methods. Genome Biol 20(1):213. 10.1186/s13059-019-1842-9 31639029 10.1186/s13059-019-1842-9 PMC6802306

[37] Uhrig S, Ellermann J, Walther T, Burkhardt P, Frohlich M, Hutter B et al (2021) Accurate and efficient detection of gene fusions from RNA sequencing data. Genome Res 31(3):448–460. 10.1101/gr.257246.119 33441414 10.1101/gr.257246.119 PMC7919457

[38] Nicorici D, Şatalan M, Edgren H, Kangaspeska S, Murumägi A, Kallioniemi O et al. (2014) FusionCatcher—a tool for finding somatic fusion genes in paired-end RNA-sequencing data. bioRxiv:011650. 10.1101/011650

[39] Xie Z, Bailey A, Kuleshov MV, Clarke DJB, Evangelista JE, Jenkins SL et al (2021) Gene set knowledge discovery with Enrichr. Current Protocols 1(3):e90. 10.1002/cpz1.90 33780170 10.1002/cpz1.90 PMC8152575

[40] Etienne B, Giraldo NA, Laetitia L, Bénédicte B, Nabila E, Florent P et al (2016) Estimating the population abundance of tissue-infiltrating immune and stromal cell populations using gene expression. Genome Biol 17(1):218. 10.1186/s13059-016-1070-5 27765066 10.1186/s13059-016-1070-5 PMC5073889

[41] Gonzalez-Galarza FF, McCabe A, Ejmd S, Jones J, Takeshita L, Ortega-Rivera ND et al (2020) Allele frequency net database (AFND) 2020 update: gold-standard data classification, open access genotype data and new query tools. Nucleic Acids Res 48(D1):D783-D78cxs8. 10.1093/nar/gkz1029 31722398 10.1093/nar/gkz1029 PMC7145554

[42] Valérie Dutoit, Christel Herold-Mende, Norbert Hilf, Oliver Schoor, Philipp Beckhove, Judith Bucher, Katharina Dorsch, Sylvia Flohr, Jens Fritsche, Peter Lewandrowski, Jennifer Lohr, Hans-Georg Rammensee, Stefan Stevanovic, Claudia Trautwein, Verona Vass, Steffen Walter, Paul R. Walker, Toni Weinschenk, Harpreet Singh-Jasuja, Pierre-Yves Dietrich (2012) Exploiting the glioblastoma peptidome to discover novel tumour-associated antigens for immunotherapy. Brain 135(4):1042–1054. 10.1093/brain/aws042 22418738 10.1093/brain/aws042

[43] Hilf N, Kuttruff-Coqui S, Frenzel K et al (2019) Actively personalized vaccination trial for newly diagnosed glioblastoma. Nature 565:240–245. 10.1038/s41586-018-0810-y 30568303 10.1038/s41586-018-0810-y

[44] Keskin DB, Anandappa AJ, Sun J et al (2019) Neoantigen vaccine generates intratumoral T cell responses in phase Ib glioblastoma trial. Nature 565:234–239. 10.1038/s41586-018-0792-9 30568305 10.1038/s41586-018-0792-9 PMC6546179

[45] Tanner M, Johanns Christopher A, Miller Connor J, Liu Richard J, Perrin Diane, Bender Dale K, Kobayashi Jian L, Campian Michael R, Chicoine Ralph G, Dacey Jiayi, Huang Edward F, Fritsch William E, Gillanders Maxim N, Artyomov Elaine R, Mardis Robert D, Schreiber Gavin P, Dunn (2019) Detection of neoantigen-specific T cells following a personalized vaccine in a patient with glioblastoma. OncoImmunology 8(4):e1561106. 10.1080/2162402X.2018.1561106 30906654 10.1080/2162402X.2018.1561106 PMC6422384

[46] Nguyen BQT, Tran TPD, Nguyen HT, Nguyen TN, Pham TMQ, Nguyen HTP et al (2023) Improvement in neoantigen prediction via integration of RNA sequencing data for variant calling. Front Immunol 14:1251603. 10.3389/fimmu.2023.1251603 37731488 10.3389/fimmu.2023.1251603 PMC10507271

[47] Xie N, Shen G, Gao W, Huang Z, Huang C, Fu L (2023) Neoantigens: promising targets for cancer therapy. Signal Transduct Target Ther 8(1):9. 10.1038/s41392-022-01270-x 36604431 10.1038/s41392-022-01270-x PMC9816309

[48] Newton Y, Sedgewick AJ, Cisneros L, Golovato J, Johnson M, Szeto CW et al (2020) Large scale, robust, and accurate whole transcriptome profiling from clinical formalin-fixed paraffin-embedded samples. Sci Rep 10(1):17597. 10.1038/s41598-020-74483-1 33077815 10.1038/s41598-020-74483-1 PMC7572424

[49] Ah-Pine F, Casas D, Menei P, Boisselier B, Garcion E, Rousseau A (2021) RNA-sequencing of IDH-wild-type glioblastoma with chromothripsis identifies novel gene fusions with potential oncogenic properties. Transl Oncol 14(1):100884. 10.1016/j.tranon.2020.100884 33074125 10.1016/j.tranon.2020.100884 PMC7569239

