# Supplementary material for: Identification and prioritisation of tumour antigen candidates from 79 glioblastoma transcriptomes
Source: Cancer Immunol Immunother. 2026 Apr 21;75(5):149. doi: 10.1007/s00262-026-04390-3 (PMC13100171; doi:10.1007/s00262-026-04390-3)
Supplement: Supplementary file 1 — Supplementary file1 (DOCX 2532 KB) [file 262_2026_4390_MOESM1_ESM.docx]

**Supplementary material**


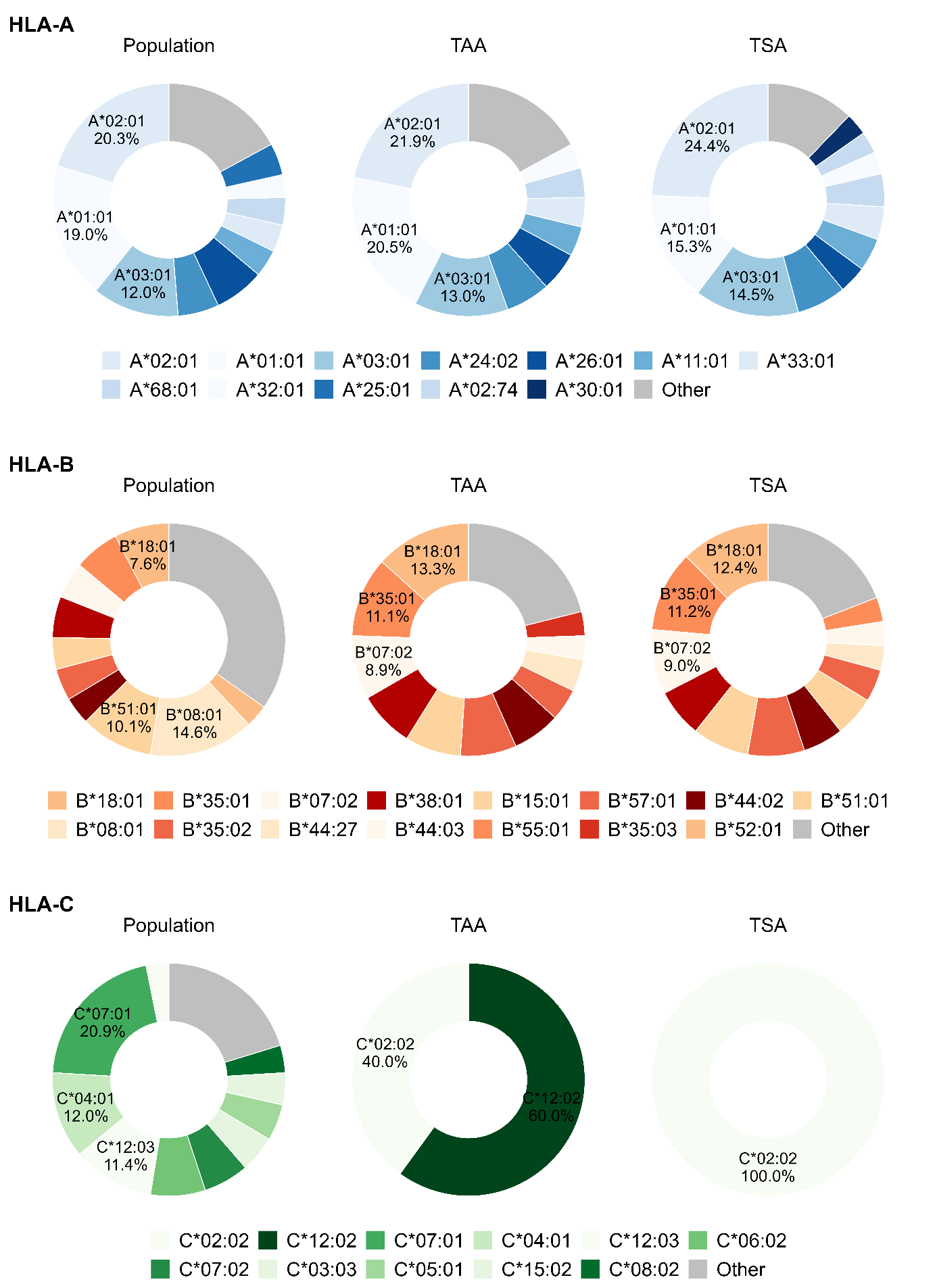


**Supplementary Figure 1** Donut plots depict the allele frequency distribution across the population (left) and among alleles actively presenting candidate peptides in the TSA (middle) and TAA (right) groups. While population frequencies were diverse across loci, only a limited subset of alleles (e.g., HLA-A*02:01, HLA-A*01:01, HLA-B*07:02) accounted for the majority of candidate peptide presentations. This highlights the skewed contribution of specific alleles to candidate peptide presentation.


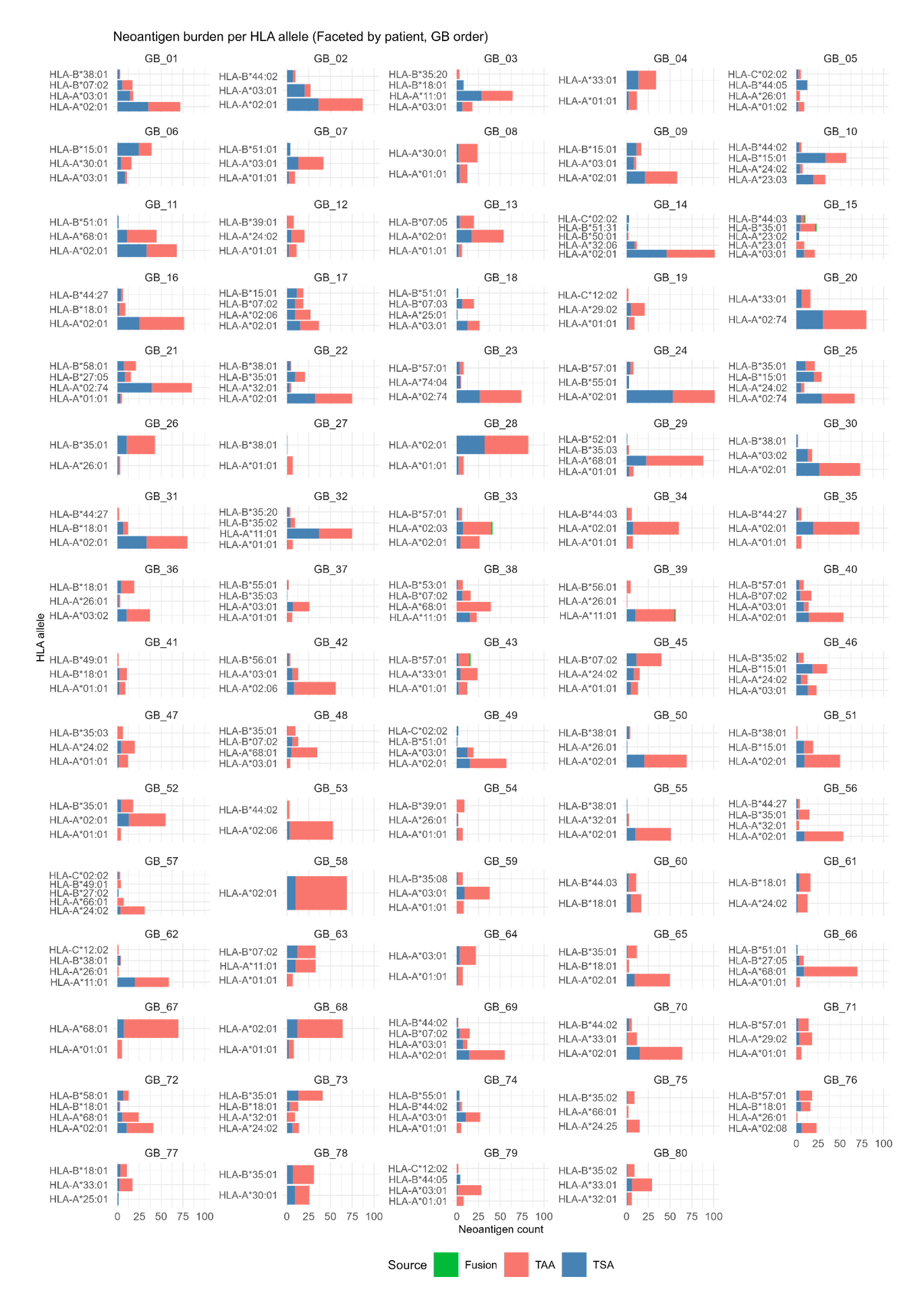


**Supplementary Figure 2** Distribution of TSAs, TAAs and fusion-derived peptides across individual samples, stratified by HLA alleles. Each panel represents a single sample, with the y-axis indicating the corresponding HLA class I alleles and the x-axis showing the number of predicted peptides. Colours denote peptide categories: green for fusion peptides, red for TAAs, and blue for TSAs. This visualization provides a per-sample overview of the predicted antigen landscape in the context of HLA presentation.


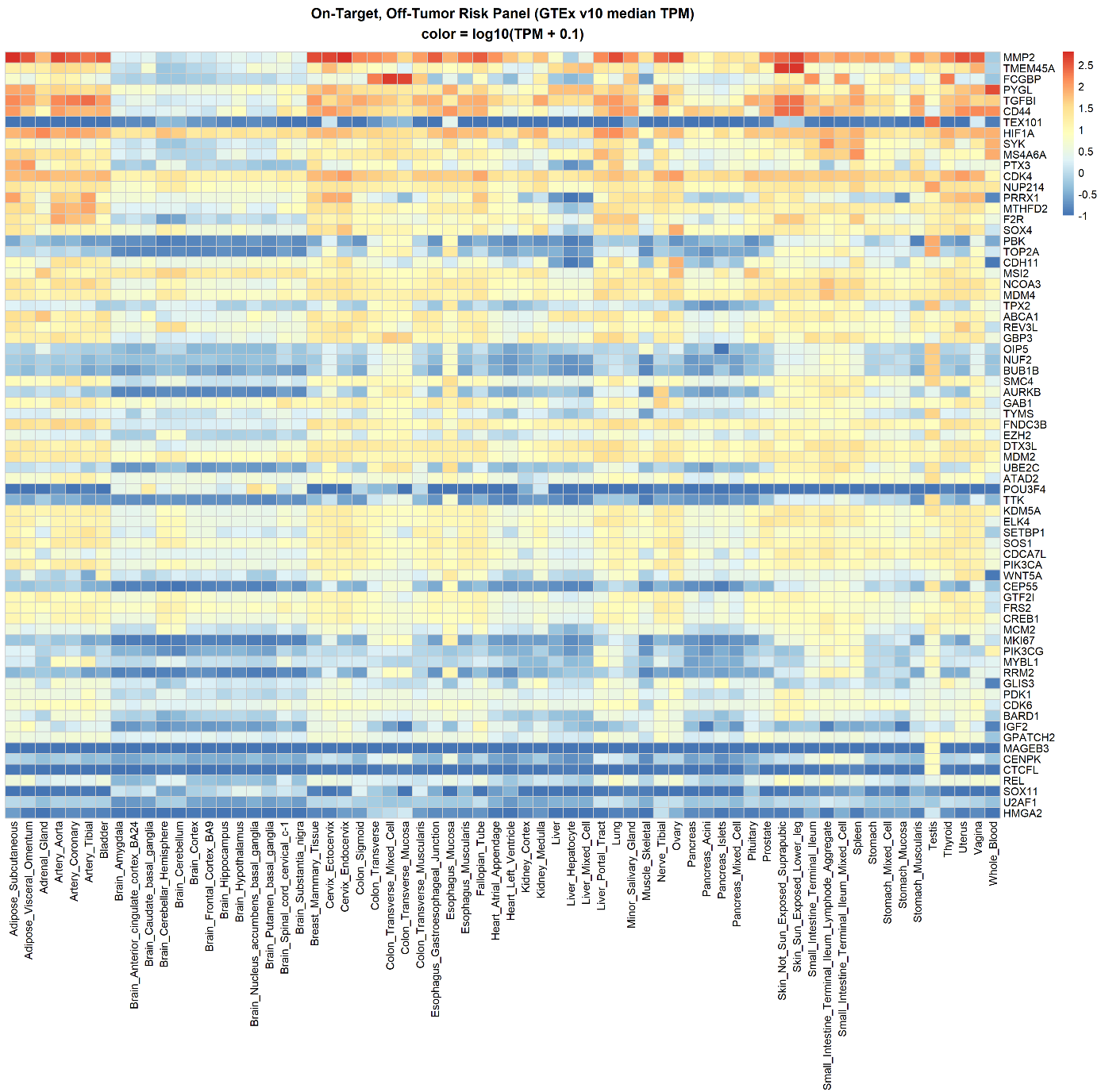


**Supplementary Figure 3** Heatmap showing the expression profiles of 71 selected genes across normal human tissues based on GTEx v10 RNA-seq data. Each column represents the median expression (TPM) for a tissue type, and each row represents one of the selected 71 genes. Expression values are log-transformed (log10[TPM + 0.1]) and colour-coded, with blue indicating low expression, yellow intermediate expression and red high expression. This visualization highlights tissue-specific versus broadly expressed genes, providing an overview of potential off-tumour expression relevant to safety assessments of therapeutic targets.

**Supplementary Table 1** Summary of RNA input quantities, PCR amplification cycles, sequencing depth, and base-calling quality metrics for GBM transcriptome libraries. PCR cycle number was determined according to library amplification performance using the Corall FFPE RNA-Seq workflow. Q30 represents the percentage of sequenced bases with Phred quality score ≥30

| **Sample** | **Input RNA (ng)** | **PCR cycles** | **Total reads** | **% Q30** |
| --- | --- | --- | --- | --- |
| **GB_01** | 200 | 13 | 37399654 | 81.38 |
| **GB_02** | 100 | 12 | 44734921 | 82.07 |
| **GB_03** | 1000 | 12 | 37429251 | 82.41 |
| **GB_04** | 1000 | 12 | 44904403 | 81.29 |
| **GB_05** | 400 | 12 | 41398718 | 83.33 |
| **GB_06** | 500 | 12 | 37319688 | 81.33 |
| **GB_07** | 100 | 13 | 40733063 | 83.39 |
| **GB_08** | 400 | 13 | 36820227 | 80.60 |
| **GB_09** | 100 | 14 | 38468121 | 81.76 |
| **GB_10** | 500 | 13 | 42454294 | 81.93 |
| **GB_11** | 400 | 12 | 51426854 | 82.20 |
| **GB_12** | 500 | 12 | 43266004 | 82.48 |
| **GB_13** | 500 | 13 | 41222684 | 82.48 |
| **GB_14** | 300 | 13 | 42343385 | 81.82 |
| **GB_15** | 500 | 11 | 42182274 | 82.39 |
| **GB_16** | 300 | 13 | 42770739 | 78.47 |
| **GB_17** | 100 | 13 | 37058744 | 83.54 |
| **GB_18** | 300 | 12 | 42004268 | 78.52 |
| **GB_19** | 500 | 11 | 34871104 | 81.46 |
| **GB_20** | 300 | 12 | 40912113 | 81.05 |
| **GB_21** | 500 | 13 | 36521160 | 81.87 |
| **GB_22** | 1000 | 13 | 46444667 | 79.80 |
| **GB_23** | 90 | 12 | 41258919 | 81.59 |
| **GB_24** | 1000 | 12 | 38314512 | 81.15 |
| **GB_25** | 300 | 13 | 37292538 | 82.17 |
| **GB_26** | 80 | 14 | 42789853 | 83.45 |
| **GB_27** | 1000 | 12 | 44254023 | 84.48 |
| **GB_28** | 500 | 12 | 37246112 | 81.01 |
| **GB_29** | 500 | 13 | 35623282 | 82.26 |
| **GB_30** | 400 | 12 | 41386563 | 76.25 |
| **GB_31** | 500 | 12 | 39490177 | 83.00 |
| **GB_32** | 1000 | 12 | 35506091 | 83.17 |
| **GB_33** | 500 | 12 | 37560972 | 85.32 |
| **GB_34** | 300 | 12 | 52083212 | 85.41 |
| **GB_35** | 500 | 12 | 54220967 | 85.21 |
| **GB_36** | 200 | 11 | 39964419 | 86.60 |
| **GB_37** | 200 | 11 | 40339897 | 86.80 |
| **GB_38** | 500 | 11 | 39366544 | 85.69 |
| **GB_39** | 300 | 11 | 45881602 | 86.84 |
| **GB_40** | 300 | 12 | 39170398 | 86.14 |
| **GB_41** | 100 | 12 | 46791566 | 83.37 |
| **GB_42** | 200 | 11 | 42114473 | 87.36 |
| **GB_43** | 300 | 11 | 43478876 | 84.66 |
| **GB_45** | 100 | 13 | 39227987 | 83.50 |
| **GB_46** | 900 | 12 | 36378408 | 82.88 |
| **GB_47** | 1000 | 11 | 43444868 | 86.18 |
| **GB_48** | 200 | 11 | 40254989 | 87.66 |
| **GB_49** | 70 | 12 | 60083477 | 86.05 |
| **GB_50** | 100 | 14 | 44264387 | 83.96 |
| **GB_51** | 1000 | 11 | 49716707 | 84.82 |
| **GB_52** | 500 | 13 | 39026877 | 82.27 |
| **GB_53** | 100 | 11 | 41060460 | 88.06 |
| **GB_54** | 100 | 12 | 40087264 | 86.04 |
| **GB_55** | 200 | 12 | 42112736 | 85.75 |
| **GB_56** | 500 | 11 | 38193352 | 85.91 |
| **GB_57** | 1000 | 11 | 47007155 | 85.28 |
| **GB_58** | 200 | 11 | 47123596 | 85.97 |
| **GB_59** | 1000 | 11 | 38602074 | 84.70 |
| **GB_60** | 300 | 11 | 40543900 | 86.36 |
| **GB_61** | 100 | 12 | 38705942 | 86.68 |
| **GB_62** | 300 | 12 | 37828779 | 85.96 |
| **GB_63** | 100 | 12 | 43459358 | 85.46 |
| **GB_64** | 200 | 11 | 39277635 | 87.37 |
| **GB_65** | 1000 | 11 | 45327321 | 85.41 |
| **GB_66** | 1000 | 11 | 39263264 | 86.17 |
| **GB_67** | 1000 | 11 | 47770668 | 86.14 |
| **GB_68** | 500 | 11 | 38599993 | 84.90 |
| **GB_69** | 1000 | 11 | 43065069 | 84.61 |
| **GB_70** | 1000 | 11 | 38432674 | 85.10 |
| **GB_71** | 1000 | 11 | 39619138 | 84.99 |
| **GB_72** | 1000 | 12 | 40849299 | 85.02 |
| **GB_73** | 500 | 12 | 44320386 | 84.08 |
| **GB_74** | 500 | 11 | 39687729 | 80.98 |
| **GB_75** | 400 | 12 | 44179682 | 85.34 |
| **GB_76** | 200 | 12 | 38854142 | 85.62 |
| **GB_77** | 500 | 11 | 41792935 | 85.21 |
| **GB_78** | 1000 | 11 | 37776651 | 85.03 |
| **GB_79** | 500 | 11 | 44596040 | 86.24 |
| **GB_80** | 500 | 12 | 43156678 | 84.92 |

**Supplementary Table 2** Genes defined as robustly overexpressed in our samples compared to normal brain cortex. CTA – cancer-testis antigens

| **Gene** | **Group** | **Gene** | **Group** | **Gene** | **Group** | **Gene** | **Group** |
| --- | --- | --- | --- | --- | --- | --- | --- |
| AURKB | oncogene | MTHFD2 | oncogene | CEP55 | CTA | GBP3 | robust |
| BUB1B | oncogene | MYBL1 | oncogene | CTCFL | CTA | GLIS3 | robust |
| CDH11 | oncogene | NCOA3 | oncogene | GPATCH2 | CTA | MCM2 | robust |
| CDK4 | oncogene | NUF2 | oncogene | MAGEB3 | CTA | MMP2 | robust |
| CDK6 | oncogene | NUP214 | oncogene | OIP5 | CTA | MS4A6A | robust |
| CREB1 | oncogene | PDK1 | oncogene | PBK | CTA | PRRX1 | robust |
| ELK4 | oncogene | PIK3CA | oncogene | TEX101 | CTA | PTX3 | robust |
| FRS2 | oncogene | PIK3CG | oncogene | TTK | CTA | PYGL | robust |
| GAB1 | oncogene | POU3F4 | oncogene | ABCA1 | robust | RRM2 | robust |
| GTF2I | oncogene | REL | oncogene | BARD1 | robust | SMC4 | robust |
| HIF1A | oncogene | REV3L | oncogene | CD44 | robust | SOX11 | robust |
| HMGA2 | oncogene | SETBP1 | oncogene | CDCA7L | robust | SOX4 | robust |
| IGF2 | oncogene | SOS1 | oncogene | CENPK | robust | TGFBI | robust |
| KDM5A | oncogene | SYK | oncogene | DTX3L | robust | TMEM45A | robust |
| MDM2 | oncogene | TOP2A | oncogene | EZH2 | robust | TPX2 | robust |
| MDM4 | oncogene | TYMS | oncogene | F2R | robust | UBE2C | robust |
| MKI67 | oncogene | U2AF1 | oncogene | FCGBP | robust | WNT5A | robust |
| MSI2 | oncogene | ATAD2 | CTA | FNDC3B | robust |  |  |

**Supplementary Table 3** Gene fusion events identified in GBM samples. The table shows fusion partner genes, type of fusion, chromosomal location, predicted protein-coding consequence and presence of frame-shifts. For fusions with potential protein-coding products, HLA allele binding predictions, median predicted binding affinities (IC50, nM), immunogenicity, stability, cleavage score and representative peptide sequences are provided. “NA” indicates cases in which the fusion is non-coding and “/” denotes unavailable data.

| **Sample** | **Fusion** | **Protein** | **Frame-shift** | **HLA allele** | **Median IC50** | **Peptide sequence** | **Immunogenicity** | **Stability** | **Cleavage** |
| --- | --- | --- | --- | --- | --- | --- | --- | --- | --- |
| **GB_15** | MYL6::ITPR2 | yes | yes | HLA-A*03:01 | 79,11 | KQYWKAKQAK | -0.23 | 0.86 | 0.78 |
|  | CTDSP2::CDK4 | yes | yes | HLA-B*35:01 | 60,91 | TAAAGNADF | 0.12 | 0.54 | 0.97 |
|  | R3HDM2::TPH2 | no | NA | / | / | / | / | / | / |
|  | TPH2::LRP1 | no | NA | / | / | / | / | / | / |
|  | R3HDM2::ARHGAP9 | no | NA | / | / | / | / | / | / |
|  | MBD6::AVIL | yes | no | HLA-B*44:03 | 98,65 | REGAVLYISY | 0.09 | 0.79 | 0.96 |
|  | MYL6B::ITPR2 | no | NA | / | / | / | / | / | / |
| **GB_20** | TMEM165::PDGFRA | yes | no | / | / | / | / | / | / |
| **GB_21** | EIF4E2::INPP5D | yes | no | / | / | / | / | / | / |
| **GB_27** | TBX20::STK17A | yes | yes | HLA-B*08:01 | 55,53 | MIITKSGSL | -0.35 | 0.08 | 0.93 |
|  | CREB5::SUN3 | yes | no | HLA-C*12:03 | 451,6 | VLMPARLRM | 0.04 | 0.19 | 0.96 |
|  | ADCY1::ABCA13 | yes | no | / | / | / | / | / | / |
|  | ST7::EIF2AK1 | yes | no | HLA-C*12:03 | 55,02 | KINDNLSTV | -0.11 | 0.06 | 0.87 |
| **GB_31** | DCTN2::CPM | yes | yes | HLA-C*05:01 | 8,73 | YADLPGITV | -0.04 | 0.14 | 0.95 |
|  |  |  |  | HLA-A*02:01 | 51,08 | FQSSNTWQI | -0.07 | 0.93 | 0.95 |
|  |  |  |  | HLA-A*02:01 | 93,59 | RLLGGSCCSI | - 0.29 | 0.90 | 0.96 |
|  |  |  |  | HLA-C*05:01 | 109,03 | YADLPGIKPV | 0.15 | 0.19 | 0.95 |
|  |  |  | no | HLA-C*12:03 | 111,11 | SSMRNLWVL | 0.19 | 0.02 | 0.80 |
| **GB_33** | GOLGA4::PLCB1 | yes | no | HLA-A*02:03 | 31,06 | LMDSTIVTPI | 0.07 | 0.72 | 0.87 |
|  |  |  |  | HLA-C*06:02 | 79,98 | YRGKYSEMI | -0.33 | 0.002 | 0.96 |
|  |  |  |  | HLA-A*02:03 | 204,79 | RLMMITVV | -0.38 | 0.94 | 0.94 |
| **GB_34** | RALA::MDM2 | no | NA | / | / | / | / | / | / |
| **GB_35** | CRACD::SRP72 | no | NA | / | / | / | / | / | / |
| **GB_36** | METAP2::ACTR6 | no | yes | / | / | / | / | / | / |
| **GB_38** | LANCL2::SEC61G | yes | no | / | / | / | / | / | / |
| **GB_39** | ANKRD13A::SLCO1B1 | yes | yes | / | / | / | / | / | / |
|  | FBXL14::CRACR2A | yes | no | HLA-A*11:01 | 412,27 | ITQLPCLKR | -0.24 | 0.65 | 0.97 |
|  | TSPAN11::CBX5 | no | NA | / | / | / | / | / | / |
|  | SLCO1B1::PIK3C2G | yes | yes | HLA-A*11:01 | 63,98 | ILYYRGWEK | 0.32 | 0.66 | 0.98 |
| **GB_40** | MLLT3::OSTF1 | yes | no | HLA-B*07:02 | 14,8 | FPRPKRGQV | -0.19 | 0.84 | 0.92 |
| **GB_41** | CCT2::MDM2 | yes | yes | HLA-C*12:03 | 41,91 | VANHHSANV | -0.06 | 0.05 | 0.55 |
|  |  |  |  | HLA-C*12:03 | 42,37 | VQYQHVCTY | -0.07 | 0.06 | 0.96 |
|  |  |  |  | HLA-C*12:03 | 59,67 | CAIPTCLYL | 0.01 | 0.01 | 0.88 |
|  | SLC16A7::MDM2 | no | NA | / | / | / | / | / | / |
|  | TIMP3::CEP95 | yes | no | / | / | / | / | / | / |
|  | AMFR::GNAO1 | yes | no | / | / | / | / | / | / |
| **GB_43** | SCAF11::NELL2 | no | NA | / | / | / | / | / | / |
| **GB_45** | CAMK1D::SKAP2 | yes | yes | / | / | / | / | / | / |
| **GB_50** | GAS7::AUNIP | yes | yes | / | / | / | / | / | / |
| **GB_51** | TTC17::FRS2 | yes | yes | / | / | / | / | / | / |
| **GB_53** | CACNB1::HMGCLL1 | yes | no | / | / | / | / | / | / |
|  | OSTM1::QKI | no | NA | / | / | / | / | / | / |
| **GB_55** | ST13::DCAF4 | yes | no | / | / | / | / | / | / |
|  | GCH1::RGS6 | yes | yes | / | / | / | / | / | / |
| **GB_57** | IFT80::PHC3 | yes | yes | HLA-B*49:01 | 285,24 | SEFNITQPEV | 0.14 | 0.46 | 0.98 |
|  | GOLIM4::TRIM59 | yes | yes | / | / | / | / | / | / |
| **GB_60** | MAGI2::GTF2I | yes | no | / | / | / | / | / | / |
| **GB_61** | RPS6KA2::SYNE1 | yes | no | HLA-C*12:03 | 291,05 | SSLSRLESM | 0.14 | 0.46 | 0.98 |
|  |  |  |  | HLA-C*12:03 | 293,02 | KVAGSVESM | -0.14 | 0.07 | 0.94 |
| **GB_62** | APOO::DMD | no | no | HLA-A*11:01 | 7,02 | IVFAQNLYK | -0.08 | 0.09 | 0.93 |
| **GB_64** | IP6K1::DOCK3 | no | NA | / | / | / | / | / | / |
| **GB_68** | CAPZA2::MET | yes | no | / | / | / | / | / | / |
| **GB_71** | SLC8A1::SOS1 | yes | yes | HLA-B*57:01 | 190,38 | LSSLMMMLF | -0.56 | 0.60 | 0.86 |
| **GB_76** | TBC1D20::CSNK2A1 | yes | yes | / | / | / | / | / | / |
| **GB_79** | CPD::SUZ12 | yes | no | / | / | / | / | / | / |

**Supplementary Table 4** Pathway over-representation analysis of 71 upregulated tumor-associated antigen (TAA) candidates. Enrichment analysis was performed using Enrichr against Reactome (2024), KEGG (2026), Gene Ontology Biological Process (2025), and MSigDB Hallmark (2020) gene sets. Statistical significance was assessed using Fisher’s exact test with Benjamini–Hochberg correction for multiple testing. Adjusted p-values are reported as false discovery rate (FDR). Gene count represents the number of overlapping TAA genes relative to the total number of genes annotated to each pathway. The top 10 significantly enriched pathways (ranked by adjusted p-value, FDR < 0.05) are shown for each database.

| **Database** | **Pathway** | **Gene Count** | **Adjusted P-value** | **Overlapping Genes** |
| --- | --- | --- | --- | --- |
| Reactome Pathways 2024 | Cell Cycle | 18/649 | 4.76x10-9 | TOP2A, NUP214, BARD1, RRM2, UBE2C, BUB1B, TYMS, SMC4, AURKB, TPX2, CDK6, CDK4, NUF2, CENPK, MDM2, OIP5, MDM4, MCM2 |
| Reactome Pathways 2024 | Cell Cycle, Mitotic | 14/516 | 9.19x10-7 | TOP2A, NUP214, RRM2, UBE2C, BUB1B, TYMS, SMC4, AURKB, TPX2, CDK6, CDK4, NUF2, CENPK, MCM2 |
| Reactome Pathways 2024 | Signaling by FGFR4 in Disease | 4/11 | 9.52x10-6 | PIK3CA, GAB1, FRS2, SOS1 |
| Reactome Pathways 2024 | Cell Cycle Checkpoints | 9/261 | 5.32x10-5 | BARD1, UBE2C, NUF2, CENPK, MDM2, BUB1B, MDM4, AURKB, MCM2 |
| Reactome Pathways 2024 | Signaling by FGFR3 in Disease | 4/22 | 9.59x10-5 | PIK3CA, GAB1, FRS2, SOS1 |
| Reactome Pathways 2024 | IRS-related Events Triggered by IGF1R | 5/52 | 9.59x10-5 | PIK3CA, GAB1, IGF2, FRS2, SOS1 |
| Reactome Pathways 2024 | IGF1R Signaling Cascade | 5/53 | 9.59x10-5 | PIK3CA, GAB1, IGF2, FRS2, SOS1 |
| Reactome Pathways 2024 | Signaling by Type 1 Insulin-like Growth Factor 1 Receptor (IGF1R) | 5/54 | 9.59x10-5 | PIK3CA, GAB1, IGF2, FRS2, SOS1 |
| Reactome Pathways 2024 | Downstream Signaling of Activated FGFR3 | 4/25 | 9.59x10-5 | PIK3CA, GAB1, FRS2, SOS1 |
| Reactome Pathways 2024 | Signaling by Erythropoietin | 4/25 | 9.59x10-5 | PIK3CA, GAB1, SOS1, PIK3CG |
| KEGG 2026 | MicroRNAs in cancer | 9/160 | 9.29x10-7 | CDK6, PIK3CA, MDM2, HMGA2, MDM4, SOS1, SOX4, CD44, EZH2 |
| KEGG 2026 | Proteoglycans in cancer | 9/197 | 2.84x10-6 | PIK3CA, MMP2, WNT5A, IGF2, MDM2, FRS2, SOS1, HIF1A, CD44 |
| KEGG 2026 | PI3K-AKT signaling pathway | 10/355 | 2.87x10-5 | CREB1, CDK6, SYK, PIK3CA, CDK4, F2R, IGF2, MDM2, SOS1, PIK3CG |
| KEGG 2026 | Pathways in cancer | 11/525 | 1.01x10-4 | CDK6, PIK3CA, CDK4, NCOA3, MMP2, F2R, WNT5A, IGF2, MDM2, SOS1, HIF1A |
| KEGG 2026 | Viral carcinogenesis | 7/185 | 1.01x10-4 | CREB1, CDK6, SYK, PIK3CA, CDK4, MDM2, REL |
| KEGG 2026 | Melanoma | 5/72 | 1.01x10-4 | CDK6, PIK3CA, CDK4, MDM2, SOS1 |
| KEGG 2026 | Kaposi sarcoma-associated herpesvirus infection | 7/195 | 1.01x10-4 | CREB1, CDK6, SYK, PIK3CA, CDK4, HIF1A, PIK3CG |
| KEGG 2026 | Glioma | 5/75 | 1.01x10-4 | CDK6, PIK3CA, CDK4, MDM2, SOS1 |
| KEGG 2026 | P53 signaling pathway | 5/75 | 1.01x10-4 | RRM2, CDK6, CDK4, MDM2, MDM4 |
| KEGG 2026 | Chronic myeloid leukemia | 5/76 | 1.01x10-4 | CDK6, PIK3CA, CDK4, MDM2, SOS1 |
| GO Biological Process 2025 | Positive Regulation of DNA-templated Transcription (GO:0045893) | 20/1274 | 1.03x10-5 | KDM5A, DTX3L, PRRX1, NCOA3, F2R, WNT5A, ATAD2, GLIS3, IGF2, HMGA2, SOX11, HIF1A, CTCFL, ELK4, CREB1, REL, SOS1, MYBL1, SOX4, GTF2I |
| GO Biological Process 2025 | Positive Regulation of Cell Population Proliferation (GO:0008284) | 12/484 | 6.19x10-5 | CDK6, CDK4, CDCA7L, F2R, WNT5A, IGF2, MDM2, HMGA2, SOX11, TTK, SOX4, EZH2 |
| GO Biological Process 2025 | Positive Regulation of Transcription by RNA Polymerase II (GO:0045944) | 16/983 | 8.58x10-5 | PRRX1, NCOA3, WNT5A, GLIS3, IGF2, HMGA2, SOX11, HIF1A, CTCFL, ELK4, CREB1, REL, SOS1, MYBL1, SOX4, GTF2I |
| GO Biological Process 2025 | Regulation of Transcription by RNA Polymerase II (GO:0006357) | 24/2250 | 1.01x10-4 | BARD1, PRRX1, NCOA3, WNT5A, GLIS3, IGF2, HMGA2, SOX11, HIF1A, AURKB, POU3F4, CTCFL, ELK4, CREB1, CDK4, MDM2, REL, MAGEB3, MDM4, SOS1, MYBL1, SOX4, GTF2I, EZH2 |
| GO Biological Process 2025 | Positive Regulation of RNA Biosynthetic Process (GO:1902680) | 12/555 | 1.07x10-4 | KDM5A, TOP2A, CREB1, DTX3L, F2R, WNT5A, ATAD2, HMGA2, MYBL1, HIF1A, SOX4, CTCFL |
| GO Biological Process 2025 | Regulation of DNA-templated Transcription (GO:0006355) | 22/2139 | 4.51x10-4 | KDM5A, BARD1, DTX3L, PRRX1, F2R, WNT5A, ATAD2, GLIS3, IGF2, HMGA2, HIF1A, POU3F4, CTCFL, ELK4, CREB1, SETBP1, MDM2, MDM4, SOS1, MYBL1, SOX4, EZH2 |
| GO Biological Process 2025 | Negative Regulation of Apoptotic Process (GO:0043066) | 10/475 | 9.39x10-4 | BARD1, CREB1, PIK3CA, F2R, WNT5A, MDM2, HMGA2, SOS1, CD44, POU3F4 |
| GO Biological Process 2025 | Negative Regulation of DNA-templated Transcription (GO:0045892) | 14/1006 | 1.25x10-3 | WNT5A, GLIS3, IGF2, HMGA2, SOX11, HIF1A, AURKB, ELK4, CREB1, MDM2, MAGEB3, MDM4, SOX4, EZH2 |
| GO Biological Process 2025 | Epithelial to Mesenchymal Transition (GO:0001837) | 4/51 | 3.58x10-3 | WNT5A, HMGA2, SOS1, HIF1A |
| GO Biological Process 2025 | Mitotic Metaphase Chromosome Alignment (GO:0007080) | 4/56 | 4.47x10-3 | NUF2, TTK, CEP55, AURKB |
| MSigDB Hallmark 2020 | G2-M Checkpoint | 13/200 | 8.26x10-12 | TOP2A, BARD1, UBE2C, TTK, MKI67, SMC4, HIF1A, AURKB, TPX2, CDK4, PBK, MCM2, EZH2 |
| MSigDB Hallmark 2020 | E2F Targets | 12/200 | 9.60x10-11 | TOP2A, BARD1, RRM2, MTHFD2, CDK4, ATAD2, BUB1B, MKI67, SMC4, AURKB, MCM2, EZH2 |
| MSigDB Hallmark 2020 | Epithelial Mesenchymal Transition | 7/200 | 8.13x10-3 | PRRX1, MMP2, WNT5A, CDH11, PTX3, TGFBI, CD44 |
| MSigDB Hallmark 2020 | Mitotic Spindle | 5/199 | 6.21x10-3 | TOP2A, TPX2, TTK, SOS1, SMC4 |
| MSigDB Hallmark 2020 | Apoptosis | 4/161 | 1.81x10-2 | TOP2A, MMP2, F2R, CD44 |
| MSigDB Hallmark 2020 | TNF-alpha Signaling via NF-kB | 4/200 | 2.18x10-2 | ABCA1, REL, PTX3, CD44 |
| MSigDB Hallmark 2020 | Apical Junction | 4/200 | 2.18x10-2 | SYK, MMP2, CDH11, TGFBI |
| MSigDB Hallmark 2020 | mTORC1 Signaling | 4/200 | 2.18x10-2 | RRM2, MTHFD2, PDK1, MCM2 |
| MSigDB Hallmark 2020 | Myc Targets V1 | 4/200 | 2.18x10-2 | CDK4, U2AF1, TYMS, MCM2 |

| **Immune cell population** | **TSA Spearman rho** | **TSA p value** | **TAA Spearman rho** | **TAA p value** | **TSA FDR BH** | **TAA FDR BH** |
| --- | --- | --- | --- | --- | --- | --- |
| T cells | 0,207 | 0,069 | -0,035 | 0,757 | 0,241 | 0,997 |
| CD8 T cells | -0,102 | 0,384 | -0,012 | 0,916 | 0,672 | 0,997 |
| Cytotoxic lymphocytes | 0,043 | 0,711 | -0,041 | 0,721 | 0,830 | 0,997 |
| NK cells | 0,297 | 0,008 | 0,084 | 0,461 | 0,058 | 0,997 |
| Monocytic lineage | -0,102 | 0,372 | -0,061 | 0,593 | 0,672 | 0,997 |
| Myeloid dendritic cells | -0,078 | 0,496 | 0,000 | 0,997 | 0,695 | 0,997 |
| Neutrophils | -0,023 | 0,841 | -0,122 | 0,284 | 0,841 | 0,997 |

**Supplementary Table 5** Association between predicted antigen burden and immune cell infiltration. Spearman correlation analysis between the number of prioritized tumour-specific antigen (TSA) and tumour-associated antigen (TAA) candidates per sample and MCP-counter–estimated immune cell populations. P-values were adjusted for multiple testing using the Benjamini–Hochberg false discovery rate (FDR) method. No statistically significant associations were observed after FDR correction

**Supplementary Table 6:** Gene-level overlap between prioritized antigen candidates and previously published GBM vaccine and neoantigen studies.

| **Gene** | **Antigen Type (this study)** | **Recurrent in This Cohort** | **Dutoit 2012** | **GAPVAC 2019** | **Keskin 2019** | **Johanns 2019** | **Latzer 2024** | **Peptide-level Match** |
| --- | --- | --- | --- | --- | --- | --- | --- | --- |
| TOP2A | TAA | Yes | Yes | No | No | No | No | No |
| CSPG4 | TSA | No | Yes | No | No | No | No | No |
| MAGI2 | TSA | Yes | No | Yes | No | No | No | No |
| RAD54B | TSA | No | No | Yes | No | No | No | No |
| SEZ6L | TSA | No | No | Yes | No | No | No | No |
| VPS13B | TSA | Yes | No | Yes | No | No | No | No |
| SMC4 | TSA | Yes | No | Yes | No | No | No | No |
| CDK4 | TAA | No | No | Yes | No | No | No | No |
| SEMA3B | TSA | No | No | No | Yes | No | No | No |
| DOCK5 | TSA | No | No | No | Yes | No | No | No |
| TFRC | TSA | No | No | No | Yes | No | No | No |
| PTEN | TSA | Yes | No | No | Yes | Yes | No | No |
| TP53 | TSA | No | No | No | Yes | No | No | No |
| GTF3C1 | TSA | Yes | No | No | Yes | No | No | No |
| UGGT1 | TSA | No | No | No | Yes | No | No | No |
| POLDIP3 | TSA | Yes | No | No | Yes | No | No | No |
| PTPN11 | TSA | No | No | No | Yes | No | No | No |
| EP300 | TSA | Yes | No | No | Yes | No | No | No |
| DSTYK | TSA | Yes | No | No | Yes | No | No | No |
| ARHGAP35 | TSA | No | No | No | Yes | No | No | No |
| NUP107 | TSA | No | No | No | No | Yes | No | No |
| EGFR | TSA | No | No | No | No | No | Yes | Yes^[[1]](#footnote-1)^ |
| DST | TSA | No | No | No | No | No | Yes | No |
| PTPRN2 | TSA | No | No | No | No | No | Yes | No |

**Supplementary Table 7:** Prior evidence of HLA class I presentation for predicted GBM antigen peptides. This table summarizes predicted TSA and TAA peptides that demonstrated exact sequence matches to entries in the IEDB MHC ligand dataset (human host, MHC class I, positive assays). “Predicted_HLA” denotes the restricting allele predicted in the present study. “Allele_concordant” indicates whether the predicted HLA allele matches an allele reported in IEDB for the same peptide.

| **Peptide** | **Predicted HLA** | **Gene** | **Source** | **Allele concordant** | **IEDB PMIDs** | **IEDB Alleles reported** |
| --- | --- | --- | --- | --- | --- | --- |
| AELWKNPTAF | B*44:02  B*44:03 | UBE2C | TAA | No  Yes | 27841757; 33912179; 35239002 | B*44:03; B*15:02; HLA class I |
| AIYDFTDTV | A*02:01  A*02:03  A*02:06  A*02:08  A*02:74 | PDK1 | TAA | No | 28228285 | A*02:07 |
| ALDGFLFVV | A*02:01  A*02:74 | NCOA3 | TAA | Yes  No | 28228285; 34087483; 35239002; 40906332 | A*02:07; HLA class I; A*02:01 |
| APRLMITHI | B*07:02 | SMC4 | TAA | Yes | 27841757; 28832583; 29508533; 29242379; 33141586; 33298915; 33936100; 35239002 | B*07:02; HLA class I |
| ATMEHHANR | A*11:01 | PDK1 | TAA | No | 31998317 | HLA class I |
| ATSVAAARK | A*03:01  A*03:02  A*11:01  A*30:01 | UBE2C | TAA | No | 28832583; 29242379 | HLA class I |
| AVIDVGINR | A*68:01 | MTHFD2 | TAA | No | 27841757; 28228285; 28832583; 29508533; 29242379; 31154438; 31998317; 35580925; 36284347; 34391887; 35239002; 36185575 | A*11:01; A*31:01; HLA class I |
| AYPFIGPFF | A*24:02 | MS4A6A | TAA | No | 35239002 | HLA class I |
| AYPFIGPFFF | A*23:03  A*24:02  A*24:25 | MS4A6A | TAA | No | 35239002 | HLA class I |
| DEIELTGIY | B*18:01 | MCM2 | TAA | Yes | 32897882 | B*18:01 |
| EAFEHPNVVR | A*68:01 | CDK4 | TAA | No | 28832583; 29242379; 35239002; 36185575 | HLA class I |
| ERYFISHVL | B*39:01 | RRM2 | TAA | No | 27841757; 28832583; 29242379; 34391887; 35239002; 36185575 | B*39:24; B*14:02; HLA class I |
| ESDFDDEAYY | A*01:01 | PBK | TAA | No | 33981976 | HLA class I |
| ETFEHPNVVR | A*68:01 | CDK6 | TAA | No | 28832583; 29242379; 35239002; 36185575 | HLA class I |
| EVPEDLAGF | A*26:01 | MKI67 | TAA | No | 34932366 | HLA class I |
| EVVDTTASTK | A*66:01 | MKI67 | TAA | No | 28832583; 29242379; 35239002 | HLA class I |
| EVVEKYEIY | A*26:01 | NUF2 | TAA | Yes | 31154438; 34932366; 35580925; 35239002; 36185575; 37759700 | HLA class I; A*25:01; A*26:01 |
| EVYGHFLGV | A*26:01 | GLIS3 | TAA | No | 36241641; 36185575 | HLA class I |
| EYGSIIAFF | A*24:02 | NUP214 | TAA | Yes | 30779550; 31154438; 35580925; 34391887; 35239002 | HLA class I; A*24:02; A*23:01 |
| FIMEGTLTRV | A*02:01  A*02:03  A*02:06  A*02:08 A*02:74 | SOS1 | TAA | Yes  Yes  No  No  No | 27841757; 28228285; 28832583; 30524438; 29242379; 29493099; 31154438; 27862975; 31998317; 33141586; 33298915; 33936100; 36010968; 35580925; 37938771; 35239002; 40906332 | A*02:01; A*02:03; HLA class I |
| FLPEAPAEL | A*02:01  A*02:06 | MCM2 | TAA | Yes  No | 28228285; 28904123; 29393594; 30524438; 29242379; 33141586; 33298915; 33912179; 32897882; 35580925; 40498839; 35239002; 40906332 | A*02:01; A*02:03; A*02:07; C*01:02; C*17:01; B*27:09; B*27:05; HLA class I; B*07:02; B*15:02; B*15:10; C*03:04; C*12:03; C*04:01 |
| FMAEIRQYI | A*02:01  A*02:03  A*02:74 | SOS1 | TAA | Yes  No  No | 33298915 | A*02:01 |
| FPDQVAIQL | B*35:02 | PYGL | TAA | Yes | 28228285; 31154438; 33141586; 33392160; 35580925; 35239002; 36185575 | B*35:01; HLA class I; B*35:02 |
| FPGFPAAAY | B*35:01  B*35:08 | MSI2 | TAA | No | 33912179; 35239002 | B*15:02; HLA class I |
| FPIEYHDIW | B*35:01  B*53:01 | RRM2 | TAA | Yes  No | 27841757; 28228285; 30779550; 33392160; 35239002 | B*35:01; HLA class I |
| FPRYNVAEI | B*07:02  B*07:03 | NUF2 | TAA | No | 33141586 | HLA class I |
| FPVFATVIL | B*35:02  B*35:03 | MCM2 | TAA | No | 28218747; 28228285; 35239002 | B*57:01; B*35:01; HLA class I |
| FSDARRLLLY | A*01:01  A*01:02 | ABCA1 | TAA | No | 36185575 | HLA class I |
| FTAPPVLGK | A*03:01 | NUP214 | TAA | Yes | 28228285; 31291378 | A*03:01; HLA class I |
| FTIDDFEIGR | A*33:01  A*66:01  A*68:01 | AURKB | TAA | No | 28832583; 29242379; 35239002 | HLA class I |
| FVFDLPIHR | A*68:01 | GBP3 | TAA | No | 28832583; 29242379; 31154438; 35239002; 36185575 | HLA class I |
| FYISWAEEY | A*29:02 | BUB1B | TAA | No | 28904123 | C*14:02 |
| GLLAKIFEL | A*02:01  A*02:06  A*02:74 | CEP55 | TAA | Yes  No  No | 28228285; 33141586; 33298915; 35239002; 40906332; 36185575 | A*02:01; A*02:04; HLA class I |
| GSFASIFWLK | A*11:01 | RRM2 | TAA | No | 35239002 | HLA class I |
| GSLSIATEK | A*11:01 | MS4A6A | TAA | Yes | 31154438; 35580925; 36284347; 37938771; 38511327; 35239002; 36185575 | HLA class I; A*11:01 |
| HEITVLIIY | B*18:01 | BUB1B | TAA | No | 36241641 | HLA class I |
| HPFHATPNTY | B*35:01 | EZH2 | TAA | Yes | 27841757; 33392160; 33912179; 35580925; 35239002 | B*35:01; HLA class I; B*15:02 |
| HPWETGHCL | B*07:02  B*07:05  B*35:01  B*35:02  B*35:03 | GPATCH2 | TAA | No | 28832583 | HLA class I |
| ILADIVISA | A*02:01  A*02:03  A*02:74 | MTHFD2 | TAA | Yes  Yes  No | 28228285; 30524438; 33141586; 33392160; 33981976; 34087483; 32897882; 36010968; 36241641; 40498839; 35239002; 40906332 | A*02:01; A*02:03; HLA class I; A*32:01; C*05:01 |
| IPRIHPNSI | B*07:02  B*07:05 | CD44 | TAA | Yes  No | 27841757; 32161166; 33298915 | B*07:02; B*51:01 |
| IYAGVGEFSF | A*23:01  A*24:02 | BUB1B | TAA | No  Yes | 28228285 | A*24:02 |
| IYTMIYRNL | A*24:02 | MDM2 | TAA | Yes | 28228285; 29242379 | A*24:02; HLA class I |
| KALDGFVMV | A*02:06 | HIF1A | TAA | No | 34391887 | HLA class I |
| KAVIDLNNRW | B*57:01  B*58:01 | U2AF1 | TAA | Yes  No | 22645359; 28218747; 28228285; 29493099; 27862975; 35580925; 35239002 | B*57:01; HLA class I |
| KFAEEFYSF | A*23:01  A*23:03  A*24:02  A*24:25 | CDCA7L | TAA | No  No  Yes  No | 28228285; 31154438; 33537174; 33141586; 33392160; 36010968; 35580925; 34185709; 34391887; 35239002 | A*24:02; HLA class I; A24 |
| KILEDVVGV | A*02:01  A*02:06 A*02:74 | TPX2 | TAA | Yes | 27841757; 28228285; 28832583; 29508533; 30524438; 29789417; 29242379; 30779550; 31222486; 27862975; 31998317; 31937595; 33141586; 33298915; 33392160; 33981976; 33936100; 34087483; 34405274; 32897882; 36010968; 35561310; 36241641; 37938771; 40498839; 34391887; 35239002; 40906332; 36185575; 41130749 | A*02:01; A*02:04; A*02:07; HLA class I; A2; C*03:04; C*12:03; A*32:01; C*05:01 |
| KLLERLPEA | A*02:01  A*02:06  A*02:74 | REV3L | TAA | No | 29242379 | HLA class I |
| KMDWIFHTI | A*32:01 | PIK3CA | TAA | No | 28228285; 29508533; 33298915; 35239002; 40906332 | A*02:07; HLA class I; A*02:01 |
| KSFPHPIDKW | B*57:01  B*58:01 | SOS1 | TAA | No | 29493099 | HLA class I |
| KSHFIVALK | A*30:01 | AURKB | TAA | Yes | 27841757; 29508533; 30524438; 29242379; 31998317; 32897882; 36581221; 35239002 | A*03:01; A*11:01; HLA class I; A*30:01; A29 |
| KSTVNGVSW | B*57:01 | NCOA3 | TAA | No | 27862975; 35239002; 36185575 | HLA class I |
| KTFARYLSF | A*32:01  A*32:06 | MCM2 | TAA | Yes | 27841757; 28218747; 29493099; 31154438; 27862975; 32897882; 35580925; 35239002 | A*32:01; B*57:01; HLA class I; C*05:01; C*14:02; B*15:17 |
| KTLAEINQKW | B*57:01 | BARD1 | TAA | Yes | 22645359; 26024233; 28228285; 29493099 | B*57:01; HLA class I |
| KVFRVENPF | A*32:01 | RRM2 | TAA | Yes | 27841757; 28832583; 32897882 | A*32:01; HLA class I |
| KVRITLVTK | A*03:01  A*30:01 | REL | TAA | No | 28832583 | HLA class I |
| LPAEVLDSL | B*35:01  B*35:02 | TGFBI | TAA | Yes | 31154438; 35580925; 35239002; 36185575 | HLA class I; B*35:01; B*35:02 |
| LPMGVVVDY | B*35:01 | NUP214 | TAA | Yes | 28228285; 31154438; 35580925; 35239002 | B*35:01; HLA class I |
| LPNGMIVTL | B*35:01  B*35:02  B*35:03 | PIK3CA | TAA | No | 29242379 | HLA class I |
| LPRRPLPPA | B*56:01 | TYMS | TAA | No | 28832583; 33298915 | HLA class I; B*07:02 |
| LTDDGDMIY | A*01:01 | HIF1A | TAA | No | 34391887 | HLA class I |
| LTSVVVTLW | B*57:01  B*58:01 | CDK6 | TAA | No | 35239002 | HLA class I |
| LYPEVFEKF | A*24:02  A*24:25 | ATAD2 | TAA | Yes  No | 27841757; 28228285; 28832583; 29242379; 30779550; 31154438; 33537174; 33392160; 36010968; 35580925; 34185709; 37938771; 34391887; 35239002; 36185575 | A*23:01; A*24:02; HLA class I; A*02:01; A24 |
| MAVEGVVFF | B*35:01 | ABCA1 | TAA | No | 31154438; 35580925 | HLA class I; C*12:03 |
| MLHDFTQQV | A*02:01  A*02:03  A*02:74 | PIK3CG | TAA | Yes  No  No | 30524438; 33298915; 35239002; 40906332 | A*02:01; HLA class I |
| MLMKNTIIAA | A*02:01  A*02:03  A*02:06  A*02:08  A*02:74 | MTHFD2 | TAA | Yes  No  No  No  No | 33298915; 40906332 | A*02:01 |
| MMVGDLLEV | A*02:01  A*02:03  A*02:74 | KDM5A | TAA | Yes  No  No | 31222486; 33141586; 33936100; 34087483; 36241641; 35239002; 40906332 | HLA class I; A*02:01 |
| NFSDLVFTY | A*29:02 | SOX11 | TAA | No | 31154438 | HLA class I |
| NIIELVHQV | A*02:06 | SYK | TAA | No | 27841757; 28832583; 29508533; 29242379; 29493099; 31154438; 27862975; 31998317; 31937595; 33141586; 33298915; 33392160; 33936100; 32897882; 35561310; 37938771; 40498839; 34391887; 35239002; 40906332; 36185575 | A*02:01; HLA class I; C*03:04; C*12:03; A*32:01; B*51:01; C*05:01 |
| NLFKWVGTI | A*02:03 | UBE2C | TAA | No | 28228285 | A*02:04 |
| NTIDRIFYL | A*02:01  A*02:06  A*02:08  A*02:74 | GAB1 | TAA | Yes  No  No  No | 36010968; 35239002 | A*02:01; HLA class I |
| NTQRSFFLR | A*33:01 | HIF1A | TAA | No | 35239002; 36185575 | HLA class I |
| NYFEVPLPY | A*29:02 | DTX3L | TAA | Yes | 27841757; 29769354; 36581221; 35239002; 36185575 | A*29:02; A29; HLA class I |
| QQVDAGLTF | B*15:01 | GAB1 | TAA | No | 35239002 | HLA class I |
| QYLDNLLVRF | A*23:03  A*24:02 | PIK3CA | TAA | No  Yes | 28228285; 36010968; 35239002 | A*24:02; A*02:01; HLA class I |
| RLMDEAKILK | A*03:01  A*03:02 | PBK | TAA | No | 29242379; 34391887 | HLA class I |
| RPFHLAAV | B*07:03 | MTHFD2 | TAA | No | 33298915 | B*07:02 |
| RPGFAAVEL | B*07:02 | SYK | TAA | No | 35239002 | HLA class I |
| RQNIGVATF | B*15:01 | SMC4 | TAA | Yes | 27841757; 28832583; 29393594; 29242379; 35561310; 36241641; 36284347; 35239002; 36185575 | B*15:01; HLA class I; B*27:09 C67S mutant |
| RRIYLILEY | B*27:05 | AURKB | TAA | Yes | 28904123; 29632046; 29393594; 29493099; 32265295; 34932366; 36241641; 35239002 | C*06:02; C*07:01; B*27:05; B*27:09; B*27:05 C67S mutant; HLA class I |
| RVHDPVTAK | A*03:01  A*03:02  A*30:01 | MTHFD2 | TAA | No | 29242379 | HLA class I |
| SAYPFIGPF | B*35:01  C*02:02 | MS4A6A | TAA | No | 35239002 | HLA class I |
| SETEDFAGF | B*18:01  B*44:02  B*44:03  B*44:27 | CDCA7L | TAA | No | 35239002 | HLA class I |
| SEYGSIIAF | B*18:01  B*35:20 | NUP214 | TAA | Yes  No | 28832583; 29242379; 30779550; 31222486; 31154438; 33981976; 34087483; 32897882; 35580925; 36241641; 31291378; 35239002; 36185575 | HLA class I; B*15:10; B*18:01; C*03:04; C*12:03 |
| SISNLVFTY | A*29:02 | SOX4 | TAA | Yes | 27841757; 29769354; 31154438; 36581221; 35239002 | A*29:02; HLA class I; A29 |
| SLDEHTSEFY | A*01:01 | MYBL1 | TAA | No | 33912179 | B*15:02 |
| SQLPPLTAV | A*02:06 | GLIS3 | TAA | No | 36010968 | HLA class I |
| SQREAAERAM | B*15:01 | MCM2 | TAA | No | 33912179 | B*15:02 |
| STTEVTQPR | A*68:01 | GTF2I | TAA | No | 29242379 | HLA class I |
| SYGYQFPGF | A*24:02  A*24:25 | MSI2 | TAA | No | 34185709; 35239002 | A24; HLA class I |
| SYIFDINTI | A*23:01  A*24:02  A*24:25 | GAB1 | TAA | No | 36010968; 37938771 | A*02:01; HLA class I |
| TEARCFYGF | B*44:03 | RRM2 | TAA | Yes | 27841757; 28832583 | B*44:03; HLA class I |
| TIIDRSFRY | A*29:02 | MKI67 | TAA | Yes | 27841757; 29769354; 33912179; 35239002 | A*29:02; B*15:02; HLA class I |
| TIMEELADA | A*02:06 | AURKB | TAA | No | 33141586 | HLA class I |
| TSLPPLPFK | A*11:01 | MKI67 | TAA | Yes | 27841757; 28228285; 29508533; 35239002 | A*11:01; A*03:01; HLA class I |
| TVYEDLRYK | A*03:01  A*11:01  A*68:01 | UBE2C | TAA | No  Yes  No | 27841757 | A*11:01 |
| TYIFAGDKF | A*24:02 | MMP2 | TAA | No | 36010968 | HLA class I |
| TYMEFLWQY | A*29:02 | NUF2 | TAA | Yes | 28904123; 29769354; 35239002 | C*07:02; A*29:02; HLA class I |
| VLMEHGVPV | A*02:01  A*02:03  A*02:06  A*02:74 | KDM5A | TAA | Yes  No  No  No | 40906332 | A*02:01 |
| VLMWEAFSY | A*29:02 | SYK | TAA | Yes | 29769354; 35239002 | A*29:02; HLA class I |
| VMHTPPVLK | A*03:01  A*03:02 | MKI67 | TAA | Yes  No | 28228285 | A*03:01 |
| VPNETIIVL | B*07:02  B*07:03  B*07:05  B*35:01  B*35:02  B*35:03  B*35:08 | MS4A6A | TAA | No  No  No  Yes  No  No  No | 31154438; 35580925; 36185575 | HLA class I; B*35:01 |
| VWSDVTPLRF | A*23:01  A*23:03  A*24:02  A*24:25 | MMP2 | TAA | Yes  No  No  No | 36010968; 35580925; 36284347; 34185709; 37938771 | A*02:01; A*23:01; HLA class I; A24 |
| VYGIRLEHF | A*24:02 | NUF2 | TAA | Yes | 28904123; 33537174; 33392160; 34932366; 36010968; 37938771; 36185575 | C*14:02; A*24:02; HLA class I |
| VYTLDIPVLF | A*23:03  A*24:02  A*24:25 | ATAD2 | TAA | No  Yes  No | 28228285 | A*24:02 |
| YGYGIRYEY | A*29:02 | PYGL | TAA | No | 36241641 | HLA class I |
| YLGGFALSV | A*02:01  A*02:74 | SYK | TAA | No | 35239002 | HLA class I |
| YLLDMPLWYL | A*02:01  A*02:06  A*02:74 | TOP2A | TAA | Yes  No  No | 28228285 | A*02:01; A*02:04; A*02:07 |
| YLNGHAETPV | A*02:03 | NCOA3 | TAA | No | 33298915; 40906332 | A*02:01 |
| YMIAHITGL | A*02:01  A*02:03  A*02:06  A*02:08  A*02:74 | TYMS | TAA | Yes  Yes  No  No  No | 27841757; 28228285; 28832583; 29508533; 30524438; 29242379; 30779550; 29493099; 31998317; 31937595; 33537174; 33141586; 33298915; 33392160; 33912179; 33981976; 33936100; 34087483; 34405274; 32897882; 36010968; 36241641; 40498839; 34391887; 35239002; 40906332 | A*02:01; A*02:03; A*02:04; A*02:07; HLA class I; B*15:02; A2; B*15:10; C*03:04; C*12:03; B*08:01; C*07:01 |
| YPFIGPFFF | B*35:01  B*35:08  B*53:01 | MS4A6A | TAA | No | 35239002 | HLA class I |
| YPGIQRTAY | B*35:01 | DTX3L | TAA | No | 33912179 | B*15:02 |
| YPLEDLPSL | B*35:01  B*35:02 | KDM5A | TAA | Yes  No | 28228285; 28832583; 29242379; 31154438; 35580925; 35239002; 36185575 | B*35:01; HLA class I |
| YQFPGFPAA | A*02:06  A*02:08 | MSI2 | TAA | No | 35239002 | HLA class I |
| YQMRPSIIFF | B*15:01 | ATAD2 | TAA | Yes | 27841757; 29393594 | B*15:01; B*27:05 C67S mutant |
| YYLSYIEEW | A*23:03  A*24:02  A*24:25 | NUP214 | TAA | No  Yes  No | 28228285; 35239002 | A*24:02; HLA class I |

1. Overlap indicates gene-level reporting in prior studies. Peptide-level match denotes identical peptide sequence and HLA restriction. *Exact EGFR peptide concordance observed with Latzer et al., 2024. [↑](#footnote-ref-1)
